# Structural basis for asymmetric bis-intercalator targeting of DNA triplex junctions enabling dual inhibition of topoisomerase I and oncogene transcription

**DOI:** 10.1093/nar/gkag520

**Published:** 2026-05-28

**Authors:** Shun-Ching Wang, Chang-Chih Hsieh, Tzu-Chun Yuan, Shan-Meng Lin, Chia-Wei Chen, Chih-Chun Chang, Shih-Chun Huang, En-Chi Wang, Yu-Jhen Huang, Ming-Hsi Chiang, Yih-Chern Horng, Ming-Hon Hou

**Affiliations:** Doctoral Program in Medical Biotechnology, National Chung Hsing University, Taichung 402, Taiwan; Graduate Institute of Genomics and Bioinformatics, National Chung Hsing University, Taichung 402, Taiwan; Institute of Chemistry, Academia Sinica, Taipei 11528, Taiwan; Graduate Institute of Genomics and Bioinformatics, National Chung Hsing University, Taichung 402, Taiwan; Graduate Institute of Genomics and Bioinformatics, National Chung Hsing University, Taichung 402, Taiwan; Department of Chemistry, National Changhua University of Education, Changhua 50058, Taiwan; Graduate Institute of Biotechnology, National Chung Hsing University, Taichung 402, Taiwan; Doctoral Program in Medical Biotechnology, National Chung Hsing University, Taichung 402, Taiwan; Graduate Institute of Genomics and Bioinformatics, National Chung Hsing University, Taichung 402, Taiwan; Department of Chemistry, National Changhua University of Education, Changhua 50058, Taiwan; Department of Chemistry, National Changhua University of Education, Changhua 50058, Taiwan; Institute of Chemistry, Academia Sinica, Taipei 11528, Taiwan; Department of Chemistry, National Changhua University of Education, Changhua 50058, Taiwan; Doctoral Program in Medical Biotechnology, National Chung Hsing University, Taichung 402, Taiwan; Graduate Institute of Genomics and Bioinformatics, National Chung Hsing University, Taichung 402, Taiwan; Graduate Institute of Biotechnology, National Chung Hsing University, Taichung 402, Taiwan; Biotechnology Center, National Chung Hsing University, Taichung 402, Taiwan

## Abstract

Achieving multi-pathway suppression with minimal toxicity remains a major goal in anticancer drug design. Here, we present the structural and mechanistic basis for asymmetric bis-intercalators QA4 and QA5, which integrate weak (quinoxaline) and strong (acridine) chromophores through optimized alkyl linkers to achieve preference recognition of non-canonical DNA triplex junctions. High-resolution crystal structures reveal a spermine-stabilized triplex junction comprising minor-groove-mediated C:G–G triplets and sheared G–G interactions, distinct from classical Hoogsteen triplexes. QA compounds engage this topology via CpG step bis-intercalation, disrupting junction integrity and inducing localized DNA deformation. QA4, with a four-carbon linker, provides optimal spacing for dual chromophore engagement, producing pronounced helical distortion. Structure-function analyses indicate that acridine-mediated DNA distortion preferentially suppresses topoisomerase I, whereas quinoxaline-induced groove deformation is correlated with transcriptional repression of multiple oncogenes, including CEACAM6. In colorectal and lung cancer xenograft models, QA4 demonstrates potent antitumor activity with negligible hepatotoxicity. These findings define the structural principles underlying topology-driven DNA recognition and establish DNA triplex junctions as druggable targets for dual-function anticancer therapeutics.

## Graphical Abstract

**Graphical Abstract:**
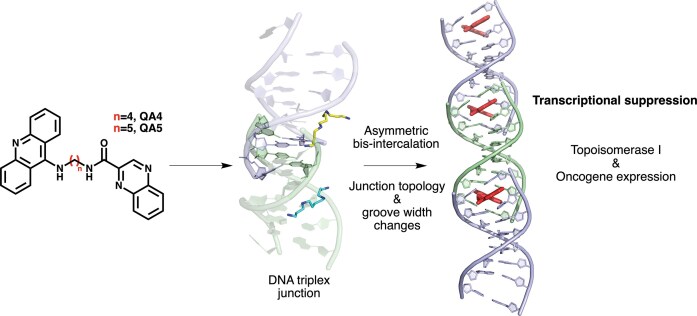

## Introduction

Cancer remains the leading cause of death worldwide, with current therapeutic approaches facing significant challenges in achieving durable clinical responses [1–3]. A major obstacle in cancer treatment is the development of drug resistance, whereby tumor cells activate compensatory signaling pathways to evade single-target therapies [4]. While combination chemotherapy regimens attempt to address this limitation by targeting multiple pathways simultaneously, they are frequently hindered by overlapping toxicities, complex dosing schedules, and unpredictable pharmacokinetic interactions between co-administered drugs [5, 6]. These challenges have motivated the development of bi-targeting agents, which are single molecules designed to modulate two distinct oncogenic mechanisms, as an alternative approach to improve therapeutic efficacy while minimizing off-target toxicity [7–9].

Current bi-targeting strategies have predominantly focused on protein targets; however, these approaches face inherent limitations, including high molecular weight, poor membrane permeability, and synthetic complexity [10]. DNA, in contrast, offers a stable and chemically accessible target for therapeutic intervention. DNA-binding agents, particularly intercalators, have demonstrated clinical success as anticancer drugs, with well-established structure–activity relationships that enable rational design. In this context, we recently reported that bis-intercalating scaffolds can target DNA junction sites and induce topological perturbations with potent antitumor activity, establishing DNA topology as a viable axis for anticancer intervention [11]. While that work demonstrated the feasibility of junction-targeting bis-intercalators, the structural basis for achieving conformational preference and multi-pathway modulation remained incompletely understood. At the same time, the therapeutic window of DNA intercalators is often narrow, constrained by the balance between efficacy and toxicity. Certain acridine derivatives have been reported to interfere with topoisomerase function, often in association with pronounced DNA distortion, although intercalation and topoisomerase inhibition are not intrinsically linked processes and depend on structural context and binding mode [12, 13]. Conversely, weak intercalators like quinoxaline derivatives demonstrate selective transcriptional repression with reduced potency [14–16]. To integrate complementary modes of action, we designed an asymmetric bis-intercalator comprising a potency-driving acridine module that imposes torsional strain on DNA to block topoisomerase I (TOP1), together with a modulatory quinoxaline module that subtly tunes groove geometry to downregulate oncogene expression [17, 18]. This asymmetric coupling strategy balances potency with selectivity by exploiting the structural plasticity of DNA.

**Figure 1.**
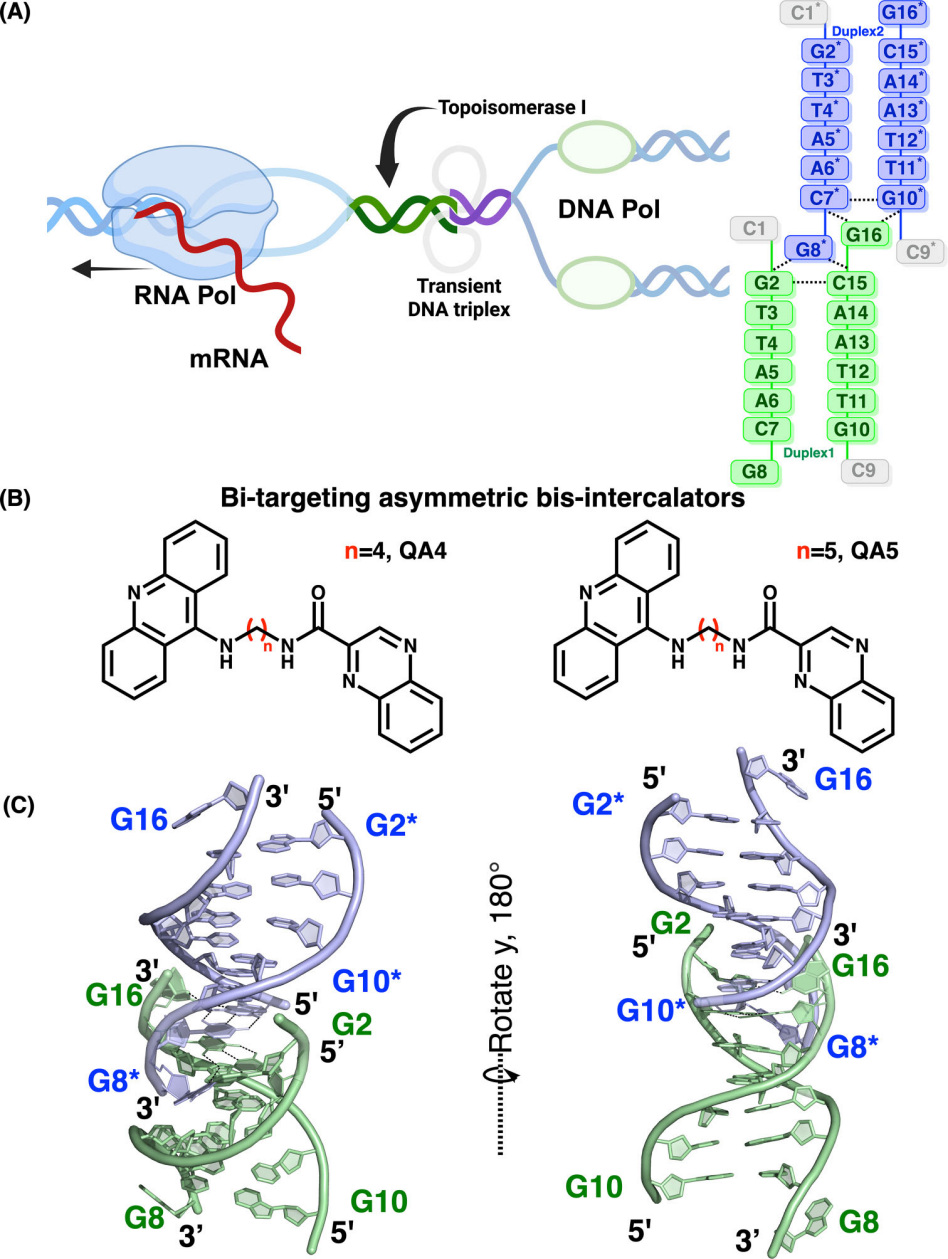
Structural features of the d(CGTTAACG)_2_ DNA. (**A**) Schematic representation of transient triplex junction formation arising in negatively supercoiled DNA behind RNA polymerase. The monomeric TOP1 is illustrated binding to canonical duplex DNA adjacent to the junction site. The triplex region is shown as a structural and topological context that may locally distort neighboring duplex segments. The nucleotide numbering scheme of the DNA structure is indicated. Created in BioRender. Wang, SC. (2026) https://BioRender.com/m91a5c8. One DNA duplex is shown in pale green, while the adjacent duplex is shown in light blue. Triplet base pairing at the triplex junction is illustrated, with dashed lines indicating hydrogen bonds and the disordered base (lacking well-defined electron density) shown in gray. (**B**) Chemical structures of the alkyl-linked asymmetric quinoxaline-acridine compounds QA4 and QA5 used in this study. (**C**) The d(CGTTAACG)_2_ crystal structure reveals continuous junctional triplet base pairing that closely mimics transient triplex junctions formed under topological stress.

Beyond base-pair sequence recognition, DNA adopts diverse higher-order structures that play critical roles in genome regulation. Non-B-DNA conformations, including G-quadruplexes, cruciforms, and triplex junctions, form transiently under topological stress generated during essential cellular processes such as transcription and replication [19, 20]. Among these structures, triplex DNA has garnered particular interest due to its formation at specific genomic loci and its involvement in regulating gene expression. Triplex junctions preferentially form in CpG-rich promoter regions where purine-rich sequences and negative supercoiling converge, creating structural intermediates that link DNA topology with transcriptional control (Fig. 1A) [21–28]. These junctions are not proposed as direct binding substrates for topoisomerases, which canonically recognize duplex DNA. Instead, triplex-associated junctions are considered structural intermediates that may locally distort adjacent duplex regions and thereby modulate topoisomerase activity indirectly. Within the nucleus, physiological polyamines such as spermine accumulate at millimolar concentrations and are known to stabilize triple-helical and junctional DNA structures by neutralizing phosphate repulsion and bridging adjacent strands under near-physiological ionic conditions [29–31]. Despite their biological significance, triplex junctions remain underexploited as therapeutic targets, primarily due to limited structural information and the absence of design principles for selective recognition. Recent advances in DNA junction chemistry have demonstrated that small molecules can selectively recognize and stabilize three-way and four-way DNA junctions, opening new avenues for structure-based drug design [32, 33]. However, the structural basis for targeting triplex junctions formed under transcription-associated torsional stress, and their potential to enable dual modulation of topological and transcriptional processes, remains unclear.

Building on these considerations, we developed QA4 and QA5, a new class of asymmetric bis-intercalators that integrate a quinoxaline and an acridine chromophore through variable-length alkyl linkers (Fig. 1B). This design is guided by three principles: (i) asymmetric coupling of weak and strong intercalators to balance efficacy and toxicity; (ii) bis-intercalation capable of engaging higher-order DNA architectures beyond canonical duplex DNA; and (iii) tunable linker geometry to optimize chromophore positioning at structurally constrained DNA sites. Using high-resolution X-ray crystallography, we characterize a spermine-stabilized, non-canonical DNA triplex junction and show that QA compounds preferentially engage and disrupt this junctional architecture through CpG step bis-intercalation. While QA compounds are expected to associate broadly with duplex DNA, our structural and biochemical analyses indicate that topologically stressed junctional DNA provides a distinct binding environment that enables localized DNA deformation and functional amplification. In this context, acridine-mediated helical distortion preferentially interferes with TOP1 activity, whereas quinoxaline-induced groove perturbation is compatible with transcriptional repression of oncogenic pathways, including CEACAM6. Together, these findings establish DNA triplex junctions as functionally privileged targets for asymmetric bis-intercalators and provide a structural framework for topology-driven, dual-function DNA-targeting anticancer agents.

## Materials and methods

### Compounds DNA oligonucleotides

The compounds QA4, QA5, QA9, QU4, and QU5 were prepared from commercially available starting materials of analytical grade. Comprehensive synthetic procedures and reaction schemes are described in the Supplementary Note. DNA oligomers purified by polyacrylamide gel electrophoresis were purchased from MDBio, Inc. in New Taipei City, Taiwan. Following procurement, the oligonucleotide solutions were carefully prepared in double-distilled water (ddH_2_O). The preparation protocol included heating the solutions to 95°C for 5 min, followed by a gradual cooling process at a rate of −0.5°C/min until room temperature was reached, which facilitated the annealing process. Quantification of the concentration (*c*) for each oligonucleotide sequence was performed using Beer’s law (*A* = ε × b × *c*). Absorbance measurements (*A*) were performed at 260 nm using an ultraviolet-visible spectrophotometer from JASCO International Co. Ltd headquartered in Tokyo, Japan. These measurements were performed in quartz cuvettes with the standard path length (b) set to 1 cm. The determination of the extinction coefficient (ε) was facilitated by the use of a special DNA calculator software known as Molbiotools.

### CD spectroscopy and thermal stability assays

Spectra were recorded at a controlled temperature of 25°C using a Chirascan™ V100 CD spectrophotometer (v4.8.3.313) in conjunction with the Pro-Data Software Suite (v4.8.3.0). A quartz cuvette with a path length of 1 mm was used for these measurements. An oligonucleotide sequence, namely d(CGTTAACG)_2_, was prepared at a concentration of 40 µM in a buffered solution of 50 mM sodium cacodylate (pH 7.3) and 5 mM magnesium chloride hexahydrate. After preparation, the mixture was heated to 95°C for 5 min in a controlled manner and then gradually cooled. The oligonucleotide samples were mixed with the compounds in various ratios, initiating incubation processes. Ellipticity spectra for circular dichroism (CD) were then recorded over the spectral range from 310–235 nm with a sampling rate of 1 s [34]. All CD spectra were recorded in triplicate for each sample, and the data were averaged before analysis. To determine the melting temperature, oligonucleotide was incubated at a concentration of 40 μM under the same buffer conditions as in the CD experiment, using a DNA-to-compound ratio of 1:6. CD melting curves were constructed by monitoring ellipticity at 276 nm while samples were exposed to a temperature gradient from 15°C–85°C at a controlled rate of 1°C per min. Each melting experiment was conducted in triplicate, and melting temperatures are reported as mean ± standard deviation (SD) derived from independent measurements.

### Crystallization

All experiments pertaining to crystallization were conducted utilizing the sitting drop vapor diffusion methodology. Crystals composed of d(CGTTAACG)_2_ DNA exclusively were cultivated through the amalgamation of a 0.6 mM oligonucleotide solution with a mother liquor comprised of 50 mM HEPES (pH 7.5), 8.5 mM CoCl_2_, 2 mM spermine tetrahydrochloride, and 5% (v/v) 2-methyl-2,4-pentanediol (MPD), subsequently equilibrated with 500 μl of 30% MPD. Incubated at 4°C, diminutive, translucent crystals materialized over the course of a few weeks. Crystals constituting the QA4–DNA complex were formed by blending 0.3 mM d(CGTTAACG)_2_ oligonucleotides with 0.6 mM QA4 within a solution containing 200 mM calcium acetate hydrate, 100 mM sodium cacodylate trihydrate (pH 6.5), and 18% PEG8000 at a 1:1 ratio, followed by equilibration with 300 μl of the mother liquor. Manifesting after 1 month at 20°C, these crystals exhibited a small, yellow, cuboidal morphology. In the case of QA5 DNA crystals, a combination of 0.3 mM d(CGTTAACG)_2_ oligonucleotides and 0.6 mM QA5 was mixed with a solution encompassing 50 mM 2-(N-morpholino)ethanesulfonic acid (MES) (pH 6.5), 200 mM potassium chloride, 5 mM hexammine cobalt (III) chloride, and 35% (v/v) MPD at a 1:1 ratio, then equilibrated with 300 μl of mother liquor. Light yellow, rod-shaped crystals emerged after ~2 weeks at 20°C.

### X-ray data collection, phasing, and structure refinement

X-ray diffraction data were acquired at BL15A1 and TPS07A of the National Synchrotron Radiation Research Center (NSRRC), Taiwan, for subsequent analysis. Data processing, reduction, integration, and scaling were conducted utilizing the HKL-2000 software package [35]. PHENIX (v1.20.1-4487) software was employed to determine the phases of the d(CGTTAACG)_2_ DNA-alone and QA4–DNA and QA5–DNA complexes via molecular replacement (phaser MR) in the *P*3_1_12, *P*2_1_ and *P*2_1_2_1_2_1_ space groups, respectively [36]. The crystallographic model of the DNA decamer (PDB ID-8W7W) and a B-form DNA duplex model created with Discovery Studio 2020 Client (v20.1.0.19295) served as initial templates for the d(CGTTAACG)_2_ DNA structure’s phase determination, subsequently aiding in phase determination for the QA4–DNA and QA5–DNA complexes. Structural refinement was carried out using PHENIX.refine package in PHENIX (v1.20.1-4487) and WinCoot (v0.9.8.92EL) [37]. Detailed crystallographic and final refinement statistics can be found in Supplementary Table S1. Final 2mF*_o_*-DF*_c_* maximum likelihood-weighted Fourier electron density maps were produced using fast Fourier transform in CCP4i and PyMOL for graphical representations of the refined structures. DNA structural parameters, such as helical parameters, torsion angles, and sugar puckers, were computed using the Curves+offline software (v2.6) [38]. Local base-pair parameters and local base-pair step parameters were determined using the 3DNA offline software (v2.4.5) [39], with corresponding values provided in Supplementary Table S2 and S3.

### Topoisomerase I and topoisomerase II activity assay

In the assay for TOP1 activity, the pHOT1 plasmid was employed as the substrate. The reaction mixture comprised freshly prepared 10x TGS buffer, consisting of 10 mM Tris–HCl (pH 7.5), 1 mM ethylenediaminetetraacetic acid, 0.15 M NaCl, 0.1% bovine serum albumin, 0.1 mM spermidine, and 5% glycerol. Additionally, 250 ng of supercoiled plasmid DNA (pHOT1), along with test compounds at six different concentrations (1, 2, 5, 10, 15, 20 μM) and TOP1 (2.5 units), were included in a final volume of 20 μl. The initial step involved incubating the mixture of supercoiled DNA and compounds at 4°C for 45 min. Subsequently, TOP1 was introduced and the reaction proceeded at 37°C for an additional 45 min. The reaction was terminated by adding a 5x stop buffer, with irinotecan serving as the positive control for comparison. Electrophoresis was conducted using a 1% agarose gel in tris–acetate–ethylenediaminetetraacetic acid (TAE) buffer without ethidium bromide. The gel was stained with ethidium bromide (0.5 μg/ml) for 15 min, destained with water for 20 min, and imaged using Uni-photo (EZ-lab) equipment. The topoisomerase II (TOP2) activity assay utilized kinetoplast DNA (kDNA) as the substrate, with relaxed circular DNA and linear kDNA employed as reference markers. The reaction mixture was prepared using a freshly concocted 5x complete reaction buffer, comprising 0.5 M Tris–HCl (pH 8.0), 1.5 M NaCl, 100 mM MgCl_2_, 5 mM dithiothreitol, 300 µg/ml bovine serum albumin, and 20 mM ATP. Into this buffer, 100 ng of kDNA, along with test compounds at six varying concentrations (1, 2, 5, 10, 15, 20 μM), and 2 units of TOP2 were added, ultimately yielding a final volume of 20 μl. The initial step involved incubating the mixture of kDNA and compounds at 4°C for 45 min. Subsequently, TOP2 was introduced and the reaction was allowed to proceed at 37°C for an additional 45 min. The reaction was terminated by adding a 5x stop buffer, and doxorubicin (DOX) served as the positive control for comparison. Following the reaction, samples underwent electrophoresis on a 1% agarose gel in TAE buffer containing ethidium bromide. Post-electrophoresis, the gel was destained in water for 20 min and subsequently imaged using Uni-photo (EZ-lab) equipment.

### DNA polymerase I activity assay

To assay the inhibition of *Escherichia coli* DNA polymerase I activity, a template (5′-TCCCCGCGCGCCCGAGCGCGCCTCCGCCCTTGCCC GCCCCCTGACGCTGCCTCA-3′) and primer (5′-TGAGGCAGCGTCAGGGGGCG-3′) derived from the promoter region of hypoxia-inducible factor 1-alpha (HIF1A) were utilized. Template-primer mixtures were prepared by heating at 95°C for 5 min in 2 µl of 10x reaction buffer (500 mM Tris–HCl, pH 7.5; 100 mM MgCl_2_; 10 mM DTT), followed by the addition of 1 µl of a dNTP mixture and ddH₂O. The mixtures were then cooled on ice to allow annealing. Next, the test compound or a clinical drug at three concentrations (10, 25, and 50 µM) was added to the annealed template-primer mixture along with DNA polymerase I (10 units) in a total reaction volume of 20 µl. The template-primer and test compound were incubated at 4°C for 45 min, followed by an additional incubation at 37°C for 20 min with DNA polymerase I. Subsequently, 7.5 µl of each reaction mixture was combined with 1.5 µl of 6x DNA loading dye and heated at 95°C for 5 min before loading onto a 12% native polyacrylamide gel in TBE buffer. Electrophoresis was performed and the gel was stained with SYBR Gold (diluted 1:20 000 in 1x TBE buffer) for 15 min, followed by destaining in water for 20 min. The gel was imaged using the DigiGel system (DGIS-10c).

### Cell culture and cell viability assay

SW620 and A549 were maintained in Dulbecco’s modified Eagle medium (DMEM) with the addition of 10% fetal bovine serum and 1% antibiotic-antimycotic solution and incubated at 37°C with 5% CO_2_. The effect of compounds on cell viability was evaluated by MTT [3-(4,5-dimethylthiazol-2-yl)-2,5-diphenyltetrazolium bromide] assay. The cells were seeded in 96-well plates with 5 × 10^3^ cells per well and incubated overnight in the medium. Cell viability was assessed at 570 nm by an enzyme-linked immunosorbent assay (ELISA) reader following exposure to compounds for 48 h at different concentrations (0.5, 1, 5, 10, 15, 20 μM).

### Cell cycle assay

SW620 cells were seeded at a density of 2 × 10^5^ cells per well into six-well plates and incubated overnight. The cells are processed for 24 h by QA4 and QA5 compounds. After treatment, the cells were fixed at −20°C with 70% EtOH. The cells were stained with a solution containing phosphate-buffered saline, propidium iodide (PI) at a ratio of 500:1, RNase A at a ratio of 200:1, and 0.05% Triton-20 for 15–30 min. The samples were analyzed for DNA content using the Attune™ NxT flow cytometer. Subsequently, the data were assessed using FlowJo V10 software.

### Tunel assay

Liver samples were fixed in 10% neutral phosphate-buffered formalin. Samples were paraffin-embedded, and liver sections were detected using an *in situ* apoptosis kit (One-step TUNEL *In Situ* Apoptosis Kit, catalog number E-CK-A320D, AMSBio Inc) according to the manufacturer’s instructions. The liver section cell nuclei were stained using a DAPI-containing mounting medium (Abcam, ab104139). Fluorescence images were captured using an Olympus IX73 inverted microscope. Four randomly chosen fields (400x magnification) were selected from each section. Manual quantification of apoptotic cells was performed using ImageJ software.

### Antitumor efficacy in the SW620 and A549 xenograft mouse model

Six to eight-week-old female BALB/C/athymic NCr-nu/nu mice were purchased from the National Laboratory Animal Center, Taiwan (Taipei, Taiwan). The experiment procedures involving live animals were approved by the Institutional Animal Care and Use Committee (IACUC) at the National Chung Hsing University (IACUC No. 109-047). SW620 (5 × 10^6^ cells) and A549 (5 × 10^6^ cells) cancer cells were injected into the right flank of the mice subcutaneously. When the tumor size reached 50–100 mm^3^, mice were divided into four or five treatment groups. For SW620 tumors, the mice were treated with the following: vehicle control [5% bovine serum albumin (BSA)], oxaliplatin (1.5 mg/kg), QA4 (1.2 mg/kg), and QA5 (1.2 mg/kg) once a week for 4 weeks. As for A549 tumors, the mice received treatment with the following: vehicle control (5% BSA), DA4 (1.2 mg/kg), DA5 (1.2 mg/kg), QA4 (1.2 mg/kg), and QA5 (1.2 mg/kg) once a week for 5 weeks via intraperitoneal injection. Mice were monitored for 4 weeks (SW620) or 5 weeks (A549), and tumor burdens and mice body weight were measured twice a week with digital calipers. Tumor volumes were calculated using the formula: length × width^2^ × 0.5. At the experiment endpoints, mice were sacrificed and then tumors and livers were for subsequent analysis.

### RNA sequencing

The cells were seeded at a density of 2 × 10^5^ cells per well and incubated overnight. After the incubation, the cells were treated with QA4 and QA5 for 16 h. Total RNA was isolated and purified from cancer cells following the protocol provided by the Trizol® manufacturer (Invitrogen, 15596026). The purity of the RNA was assessed using a NanoDrop spectrophotometer. RNA degradation and integrity were assessed using the Qsep 100 DNA/RNA Analyzer (BiOptic Inc., Taiwan). The complementary DNA libraries were assessed for quality using the Qubit® 2.0 Fluorometer (Thermo Scientific) and Agilent Bioanalyzer 2100 system. The data obtained through high-throughput sequencing (Illumina NovaSeq 6000 platform) underwent a filtering process to remove low-quality reads. The resulting high-quality data were then utilized for subsequent analysis. Differential gene expression, including Disease Ontology (DO) pathway enrichment, identify enriched biological functions and activated pathways from the molecular signatures database (MSigDB).

### Real-time quantitative reverse transcription PCR

Cells were seeded at a density of 2 × 10^5^ cells per well and incubated overnight. After incubation, the cells were treated with 10 μM of the respective compounds for 16 h. Total RNA was then extracted from SW620 colorectal cancer cells using Trizol reagent (BIO-CHECK LABORATORIES LTD). A 0.5 μg RNA aliquot was reverse transcribed using EntiLinkTM Reverse Transcriptase (ANTEC-bioscience. Inc). The isolated messenger RNA (mRNA) samples were analyzed by reverse transcription-polymerase chain reaction (RT-PCR) with the following primer pairs: carcinoembryonic antigen-related cell adhesion molecule 6 (CEACAM6) (forward primer: 5ʹ-TCAGCCACTGGCCTCAATAG-3ʹ, reverse primer: 5ʹ- TCTGGTCCAATCTGCCAGTC- 3ʹ). To standardize the mRNA levels of the individual target genes, the expression levels of the housekeeping gene glyceraldehyde-3-phosphate dehydrogenase (GAPDH) were determined in parallel for each sample. The relative gene expression of CEACAM6 was determined using SYBR Green dye (ANTEC-bioscience. Inc) with the Step One Real-Time PCR System (Applied Biosystems). All amplification reactions were performed for 40 cycles and in duplicates (a hold phase of 10 min at 95°C, followed by a cycle phase of 10 s at 95°C, 30 s at 60°C, 15 s at 95°C, 15 s at 55°C, and 15 s at 95°C). The data were normalized against the GAPDH expression values. The relative expression level of each target gene was calculated according to the relative quantification threshold cycle (Ct) method: $\Delta C{\mathrm{t}} = C{\mathrm{t}}( {{\mathrm{target\ gene}}} ) - C{\mathrm{t}}( {{\mathrm{GAPDH}}} )$, $\Delta \Delta C{\mathrm{t}} = {\mathrm{\Delta }}C{\mathrm{t}}( {{\mathrm{target\ gene}}} ) - {\mathrm{\Delta }}C{\mathrm{t}}( {{\mathrm{GAPDH}}} )$, and ${\mathrm{relative\ quantification}} = 2^\wedge - \Delta \Delta C{\mathrm{t}}$.

### Cell apoptosis analysis

SW620 cells were seeded in six-well plates at a density of 2 × 10^5^ cells per well and cultured overnight in DMEM (pH 7.2) under standard conditions. Cells were then treated with fresh DMEM containing the indicated compounds at a final concentration of 50 μM for 24 h, while untreated cells were included as controls. Following treatment, cells were collected and stained with an Annexin V-fluorescein isothiocyanate (FITC)/PI apoptosis detection kit (Thermo Fisher Scientific) according to the manufacturer’s instructions. After incubation for 15 min at room temperature in the dark, apoptotic cell populations were analyzed by flow cytometry. Early and late apoptotic cells were distinguished based on Annexin V-FITC and PI staining profiles. Data acquisition was performed using an Attune™ NxT flow cytometer, and data analysis was conducted using FlowJo V10 software.

## Results

### Crystal structure of d(CGTTAACG)_2_ reveals a non-canonical triplex junction with minor groove-mediated triplet base pairing

Based on the rational design of QA compounds, triplex DNA intermediates formed under topological stress were considered relevant structural targets. To visualize such non-canonical DNA architectures, we designed the d(CGTTAACG)_2_ DNA sequence containing terminal CpG steps flanked by AT-rich regions, a sequence context proposed to facilitate experimentally triplex junction formation [40]. The crystal structure was solved at a resolution of 1.61 Å (Supplementary Table S1). Crystallization was achieved in the presence of spermine as a polyamine component of the crystallization condition, which facilitates lattice formation and stabilizes higher-order DNA architectures. The oligonucleotide self-assembles into an antiparallel duplex comprising two continuous independent duplexes in one asymmetric unit. These duplexes form an atypical triplex junction at the terminal CpG steps, representing a non-canonical DNA architecture distinct from classical major groove-mediated triplexes. This structure provides a structural model for triplex junctions that may arise under transcription-associated topological stress (Fig. 1C). To facilitate discussion, duplexes are labeled as Duplex 1 and Duplex 2. The oligonucleotides in Duplex 1, on one strand, were numbered from C1 to G8 and those on the complementary strand were numbered from C9 to G16 in the 5′→3′ direction, while those in Duplex 2 were denoted with an asterisk (*) (Fig. 1A, right panel).

**Figure 2.**
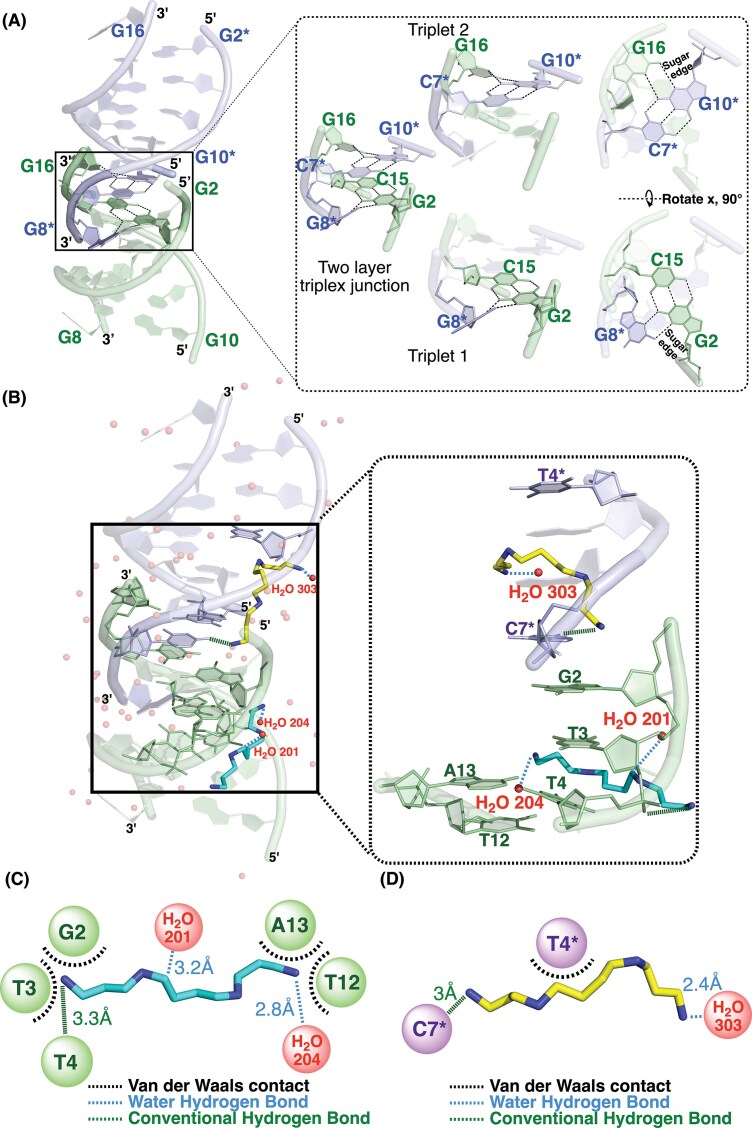
Comprehensive junction site analysis of the interactions within the d(CGTTAACG)_2_ DNA structure. (**A**) Overall view of the ligand-free DNA structure showing the base pairing interactions at the triplex junction. The magnified view highlights two stacked C:G–G triplet layers stabilized by minor groove-associated, non-canonical sheared G–G base pairing. The first layer contains G16 from one duplex interacting side by side with the C7*:G10* base pair from the adjacent duplex, whereas the second layer contains G8* from one duplex engaging with the G2:C15 base pair from the other. The guanine residues in each triplet exhibit pronounced propeller twisting (33.5° for G16-G10* and −34.5° for G8*-G2), forming a non-coplanar stacking geometry that promotes sugar-edge-mediated hydrogen bonding. Both side and top views are shown. (**B**) Interactions between DNA and spermine. The DNA is shown as a cartoon with base pairs in stick representation. Two spermine molecules (cyan and yellow) bridge the duplexes through hydrogen bonding and van der Waals contacts. Hydrogen bonds are shown as dashed lines (green for conventional, blue for water-mediated). Water molecules (red spheres) further stabilize the contact network. A magnified view of the junction highlights the detailed spermine interactions: one spermine (cyan) forms hydrogen bonds with G2, T3, T4, T12, A13, and water molecules, whereas the other (yellow) interacts with C7*, T4*, and a water molecule at the adjacent duplex interface. Spermine was included as a polyamine component of the crystallization condition. (**C**, **D**) Summary of van der Waals contacts, water-mediated hydrogen bonds, and conventional hydrogen bonds observed at the junction site.

The junction is characterized by two layers of triplet base pairing formed within the C1G2/C15G16 region of Duplex 1 and the C7*G8*/C9*G10* region of Duplex 2, where C1 and C9* are absent (Fig. 1C and Supplementary Fig. S1). In this non-canonical triplex architecture, the third-strand bases approach from the minor groove side and form C:G–G triplet base pairings that involve direct hydrogen bonding between the sugar edges of guanine residues. Specifically, G16 and G8* from the third strands interact with the G10* and G2, respectively, through hydrogen bonds formed between the N2 and N3 atoms of their sugar edges. These interactions represent a non-canonical sheared G–G base pairing arrangement that establishes side-by-side sheared G:G pairing, defining a minor groove-associated triplet configuration distinct from both Hoogsteen and reverse Hoogsteen geometries. The guanine bases within each C:G–G triplet exhibit pronounced propeller twisting, indicating a non-coplanar stacking geometry that promotes sugar-edge-mediated hydrogen bonding. Structural analysis using the Web-3DNA server revealed propeller twist angles of ~33.5° for G16-G10* and −34.5° for G8*-G2, consistent with a substantial torsional displacement within each triplet layer. This arrangement produces two stacked C:G–G layers that generate a compact triplex junction stabilized by spermine-mediated electrostatic bridging. Triplet 1 is formed by the G8* base of Duplex 2 engaging the G2:C15 Watson–Crick pair of Duplex 1 from the minor groove side. In contrast, Triplet 2 involves G16 in Duplex 1 interacting with the C7*:G10* Watson–Crick pair of Duplex 2 (Fig. 2A). The junction is further stabilized by two spermine molecules that bridge base edges and phosphate backbones through hydrogen bonding and van der Waals contacts (Fig. 2B). One spermine molecule interacts with G2, T3, T4, T12, and A13, and the other with C7* and T4* (Fig. 2C and D). Polyamines such as spermine are known to stabilize triple-helical DNA architectures by neutralizing phosphate repulsion and bridging adjacent strands. In the present structure, spermine-mediated interactions contribute to stabilization of the triplex junction [30, 31].

Collectively, the d(CGTTAACG)_2_ DNA structure adopts a non-canonical triplex junction conformation stabilized by minor groove-associated C:G–G triplet base pairing, in which non-canonical sheared side-by-side G–G interactions generate a compact and well-ordered junction core. This distinctive architecture captures structural features consistent with those proposed to arise in genomic DNA under transcription-induced topological stress, providing an effective structural framework for subsequent analyses of how DNA junction architectures may influence topoisomerase activity and transcriptional regulation.

### Intercalation of QA compounds induces a structural disassembly of the d(CGTTAACG)_2_ triplex junction

**Figure 3.**
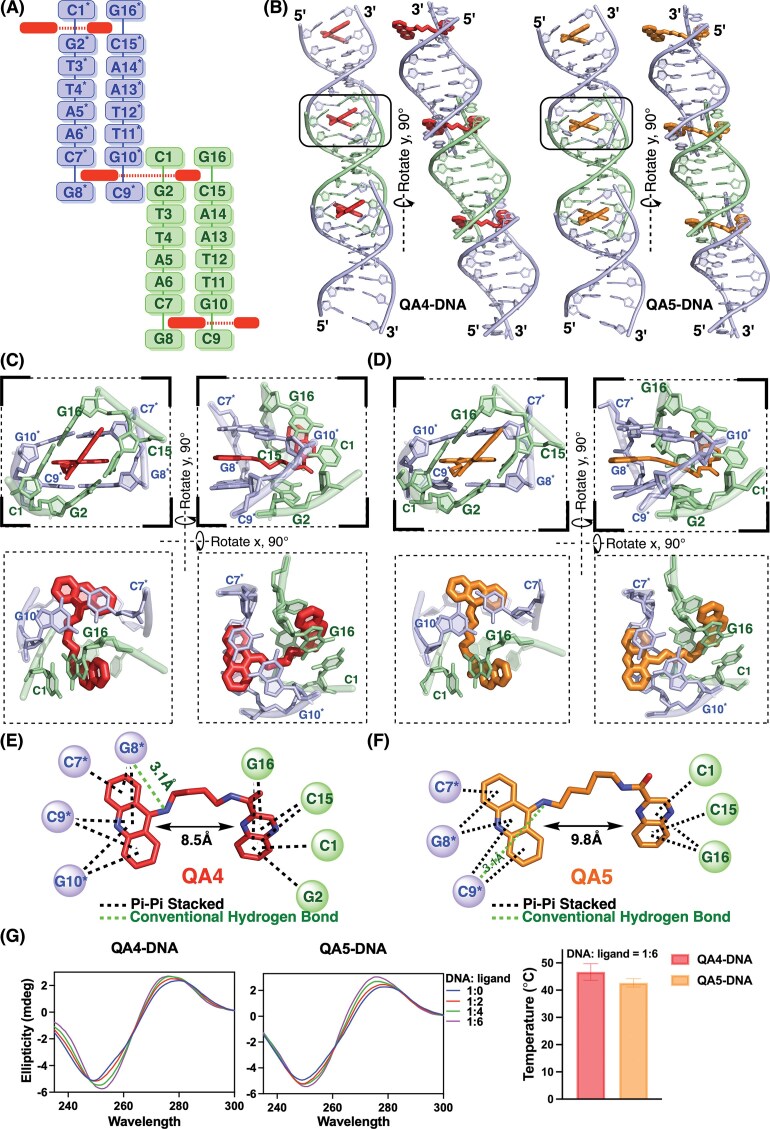
Intercalation and stabilizing effects of QA4 and QA5 on d(CGTTAACG)_2_. (**A**) Schematic representation and numbering scheme of the crystal structures of two asymmetric bis-intercalator complexes. One DNA duplex is shown in pale green, while the other adjacent duplex is shown in light blue. The intercalation of QA4 or QA5 (shown by two red rectangles connected by a dashed line, with the smaller rectangle representing the quinoxaline chromophore and the larger rectangle representing the acridine chromophore) at the CpG sites in each duplex mediates contact between adjacent duplexes by intercalating. The enlarged spacing observed between certain terminal base pairs reflects intercalation-induced expansion of the base-pair step (increased helical rise). (**B**) QA4–DNA (left) and QA5–DNA (right) complexes showing the QA4- and QA5-induced bis-intercalation of DNA structure. (**C**, **D**) magnified views of the intercalation sites in QA4–DNA and QA5–DNA complexes. The intercalated QA4 (red sticks) and QA5 (orange sticks) are shown at the C1pG2/C15pG16 step of one DNA duplex and the C7*pG8*/C9*pG10* step of the adjacent duplex. Both complexes are visualized from front, top, and side perspectives to illustrate the spatial orientation and base-stacking interactions at the intercalation sites. (**E, F**) The total number of π–π stacking and conventional hydrogen bonding interactions of QA4 and QA5 is shown. The numbers are used to represent the distance, where the unit is Å. CD spectra demonstrating the stabilization of d(CGTTAACG)_2_ upon QA4 and QA5 binding. (**G**) CD spectra of d(CGTTAACG)_2_ in the presence of different ratios of QA4 and QA5. Spectra were recorded in the presence of 40 μM oligonucleotides prepared in a buffer containing 50 mM sodium cacodylate (pH 7.3) and 5 mM magnesium chloride. Right panel: CD-monitored thermal denaturation profiles of d(CGTTAACG)_2_ (20 μM) in the presence of QA4 or QA5 at a DNA:ligand ratio of 1:6. Apparent midpoint transition temperatures were determined from the half-transition between low- and high-temperature baselines and are reported as mean ± SD (*n* = 3). This ratio was chosen as CD spectra exhibited saturation at 276 nm after the addition of six equivalents of each compound.

To investigate the binding mechanism of QA4 and QA5 to d(CGTTAACG)_2_ at the atomic level, we determined the crystal structures of QA4 and QA5 complexed with d(CGTTAACG)_2_. These structures, denoted as QA4–DNA and QA5–DNA complexes, were resolved at 1.93 and 1.27 Å resolution, respectively (Supplementary Table S1). In both structures, each asymmetric unit contained a single DNA duplex intercalated by one ligand (Supplementary Figs S2 and  S3). The oligonucleotides on one strand were numbered from C1 to G8 and those on the complementary strand were numbered from C9 to G16 in the 5′→3′ direction, where the quinoxaline moiety intercalated at the terminal C1pG2/C15pG16 step. Interestingly, the acridine moiety of the same QA compound intercalated into the C7*pG8*/C9*pG10* step of a symmetry-related complex, in which the oligonucleotides were denoted with an asterisk (*) (Fig. 3A and B). This arrangement positions the two chromophores at sites corresponding to the original triplex junction interface of the d(CGTTAACG)_2_ assembly, consistent with a ligand-associated conversion of the triplex junction into two separated duplexes. Detailed structural analyses reveal that both the quinoxaline and acridine moieties of QA4 stack well with four bases, C1, G2, C15, and G16 for quinoxaline, and C7, G8, C9*, and G10* for acridine, within the intercalation site (Fig. 3C and E). In contrast, the two moieties of QA5 form stacking interactions with only three bases, C1, C15, and G16 for quinoxaline, and C7*, G8*, and C9* for acridine (Fig. 3D and F). These observations indicate more extensive stacking stabilization for QA4. To assess whether these structural differences translate into altered DNA conformational behavior in solution, CD spectroscopy was performed. In the absence of ligand, the CD spectrum exhibited a negative peak near 250 nm and a positive peak around 280 nm. Upon titration with QA4 or QA5, the ellipticity at ∼250 nm decreased, and the positive band shifted from 280 to 276 nm, consistent with ligand-induced conformational rearrangement (Fig. 3G, left panel). Moreover, the presence of an isodichroic point near 260 nm suggests a two-state equilibrium between free DNA and ligand-bound DNA, implying that intercalation of QA compounds induces a well-defined structural rearrangement rather than multiple intermediate conformations [41, 42]. Consistent with the π–π stacking interaction patterns, CD-monitored thermal denaturation analysis revealed ligand-dependent differences in apparent thermal stability. The QA4–DNA and QA5–DNA complexes exhibited midpoint transition temperatures of 46.7 ± 3.1°C and 42.7 ± 1.5°C, respectively (mean ± SD, *n* = 3) (Fig. 3G, right panel). The higher transition temperature observed for QA4 aligns with its more extensive stacking interactions and more favorable junctional accommodation, attributable to the four-carbon linker, which facilitates simultaneous engagement of both chromophores at the junction site. Additionally, density functional theory calculations reveal a different free energy difference for the conformational transition from a free linear form to a bent conformation for QA4 and QA5, ~0.49 kcal/mol and 5.14 kcal/mol in solution, respectively (Supplementary Fig. S4). These calculations indicate that QA4 has a lower energetic barrier for adopting a bent conformation in solution, which may facilitate its accommodation within the constrained geometry of the junctional binding site. Collectively, these results provide structural insights into ligand-associated disruption of triplex junction architectures and highlight the importance of linker length in asymmetric bis-intercalator design.

### Structural impact of QA binding within junctional DNA architecture

**Figure 4.**
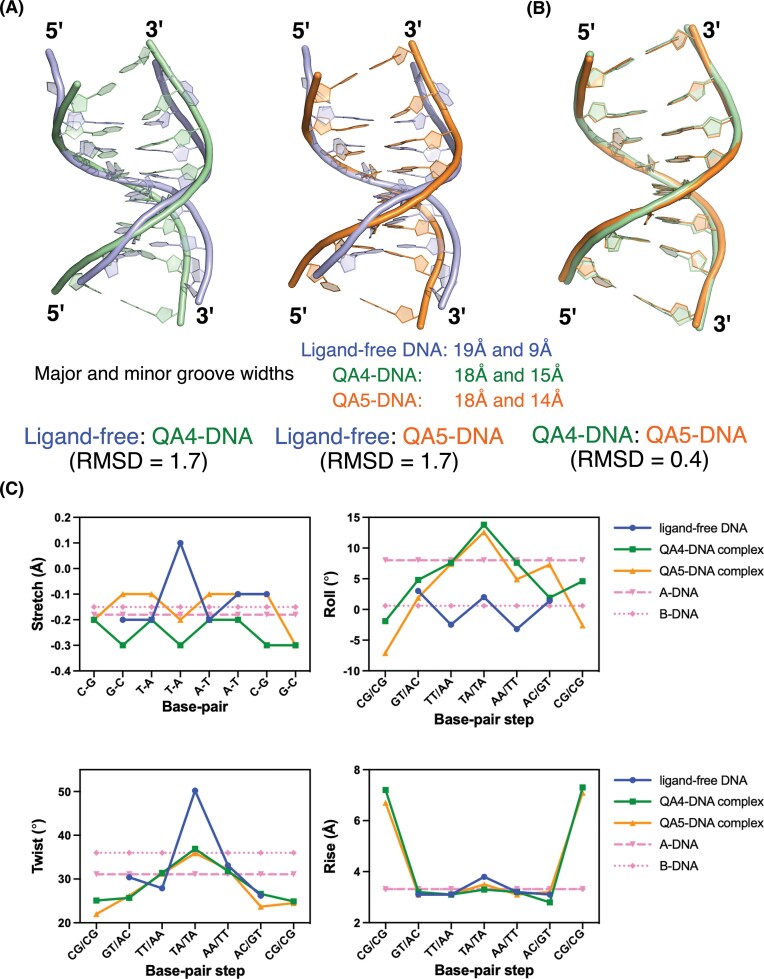
Comparison of the DNA structures in QA4–DNA and QA5–DNA complexes with d(CGTTAACG)_2_ ligand-free DNA. (**A**) Superimposition of the crystal structures of ligand-free DNA with QA4–DNA complex and QA5–DNA complexes, showing the overlap between these structures. The RMSD for the same number of DNA atoms for these structures is 1.7 Å, indicating structural perturbations upon ligand binding. The impact of ligand intercalation on the central T_4_A_5_/T_12_A_13_ base-pair step was evaluated by measuring the major and minor groove widths, which were defined as interstrand P-P distances, using Web 3DNA 2.0 before and after compound binding. (**B**) The superimposition of the QA4–DNA and QA5–DNA complex structures shows an RMSD of 0.4 Å, indicating localized structural variations despite their overall conformational similarity. (**C**) The variations in the DNA base pair parameter stretch distances, the DNA base pair step parameters roll, twist angles, and rise distances observed in these structures are shown. The values of the DNA alone structure and the standard A- and B-DNA are given for reference.

To assess the detailed structural impact of QA4 and QA5 intercalation on each DNA duplex, we superimposed each QA–DNA complex with its symmetry-related counterpart (QA–DNA*) (Supplementary Fig. S5A). Additionally, we aligned the QA4–DNA and QA5–DNA structures, as well as each complex with the ligand-free DNA, and calculated the RMSD (root mean square deviation) values. Structural alignment of the two asymmetric units showed an identical conformation. The RMSD between each QA–DNA complex and ligand-free DNA was 1.7 Å (Fig. 4A), whereas the RMSD between the QA4–DNA and QA5–DNA complexes was 0.4 Å (Fig. 4B). These results indicate that the overall DNA conformational change induced by QA4 and QA5 is structurally conserved. On the other hand, structural alignment at the quinoxaline and acridine intercalation sites revealed significant local differences at the quinoxaline and acridine intercalation sites, consistent with chromophore-dependent conformational variation (Supplementary Fig. S5B and C). To further characterize drug-induced conformational changes, we comprehensively analyzed DNA parameters and compared them with those of the ligand-free DNA structure and standard A- and B-DNA values. The mean helical twist (h-twist) of the ligand-free DNA was 33.6° ± 9.7°, whereas the values for the QA4–DNA and QA5–DNA duplexes were 28.9° ± 4.6° and 28.0° ± 5.2°, respectively. Of all the base pair steps, the most pronounced change was observed at the central T4A5/T12A13 steps, where the twist angles significantly decreased upon ligand binding, measuring 36.9° for QA4–DNA and 35.9° for QA5–DNA, in contrast to 50.2° in the ligand-free DNA. These reduced twist angles indicate helical unwinding upon ligand intercalation (Supplementary Table S3). The average helical rise (h-rise) values increased by ~2.0 Å in the ligand-bound complexes, reflecting an elongated conformation commonly observed in DNA intercalation complexes. Additionally, the ligand-free DNA structure exhibited roll angles typical of B-DNA (0°), whereas ligand binding increased roll angles toward an A-DNA-like geometry (Fig. 4C). The cumulative roll angles of the central five base pair steps were 0.7°, 35.7°, and 34.1° in ligand-free DNA, QA4–DNA, and QA5–DNA, respectively. Consistently, the QA–DNA complexes displayed a narrower major groove and a wider minor groove compared to the ligand-free DNA (Fig. 4A). These groove width changes are consistent with a DNA bending geometry oriented toward the major groove. Moreover, the stretch parameter at the central T:A base pair shifted from a positive value (0.1 Å in ligand-free DNA) to negative values (−0.3 Å in QA4–DNA and −0.2 Å in QA5–DNA), indicative of increased DNA rigidity commonly observed in A-DNA (Fig. 4C). These observations indicate that QA4 and QA5 induce DNA conformational changes, including unwinding, elongation, and backbone bending, with QA4 causing more pronounced structural perturbations, consistent with its enhanced stacking and stabilizing effects.

### QA compounds suppress TOP1-mediated DNA relaxation and modulate oncogene transcription

**Figure 5.**
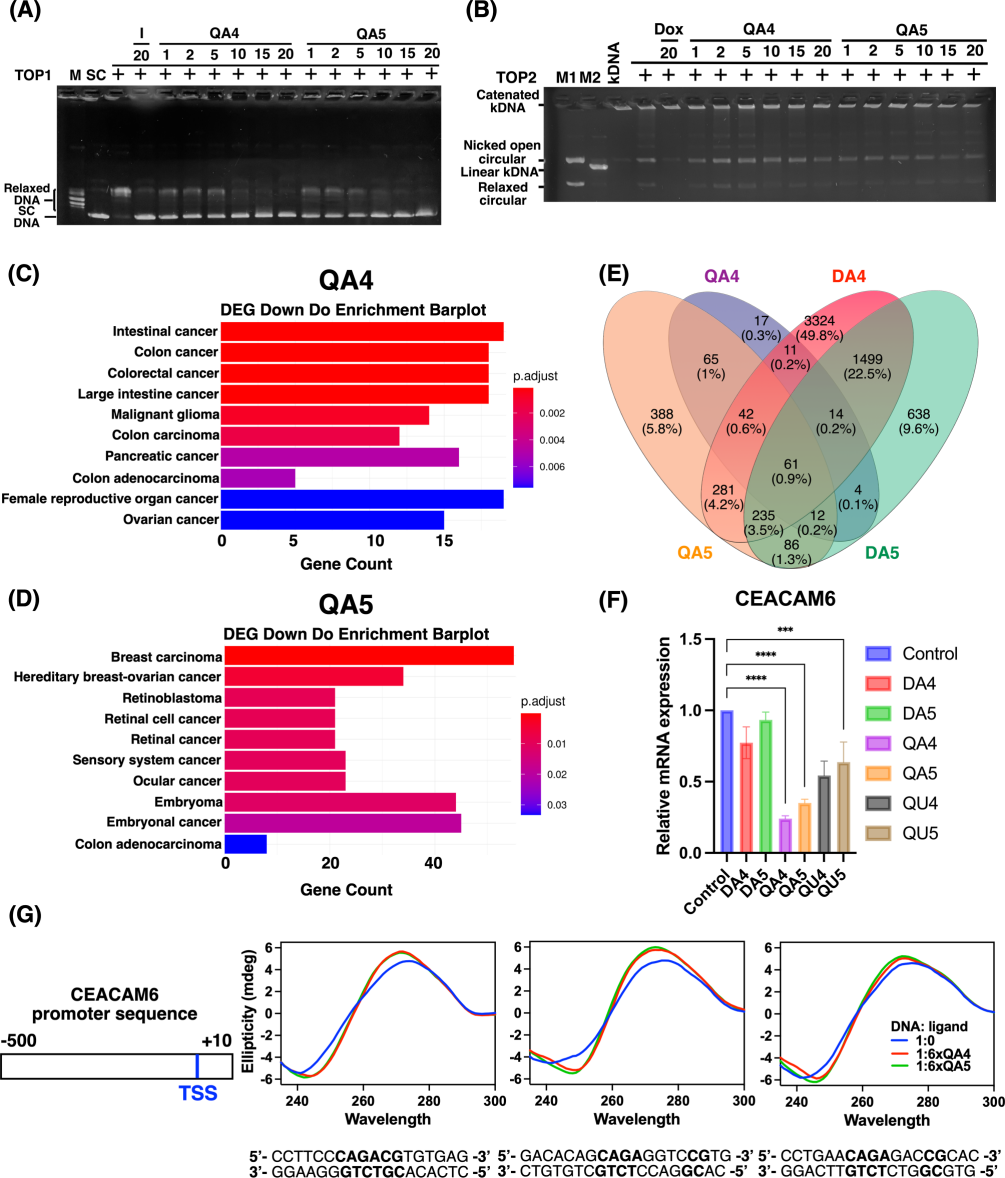
Bi-targeting asymmetric bis-intercalators suppress TOP1 and transcription processes. (**A**) Representative gel for the TOP1 activity assay. Lane 1, marker; lane 2, supercoiled DNA (pHOT1 plasmid); lane 3, pHOT1 DNA and TOP1; lane 4, pHOT1 DNA, TOP1, and 20 µM irinotecan; lanes 5–10, supercoiled DNA, TOP1, and QA4 at 1, 2, 5, 10, 15, and 20 µM, respectively; and lanes 11–16, supercoiled DNA, TOP1, and QA5 at 1, 2, 5, 10, 15, and 20 µM, respectively. (**B**) Representative TOP2 activity assay gel. Lane 1, decatenated kDNA (marker 1); lane 2, linearized kDNA (marker 2); lane 3, kDNA alone; lane 4, kDNA and TOP2; lane 5, kDNA, TOP2, and 20 µM Dox; lanes 6–11, kDNA, TOP2, and QA4 at 1, 2, 5, 10, 15, and 20 µM; and lanes 12–17, kDNA, TOP2, and QA5 at 1, 2, 5, 10, 15, and 20 µM. (**C**, **D**) DO enrichment analysis of differentially expressed genes (DEGs) following QA4 and QA5 treatment, with colors representing adjusted *P*-values (p.adjust). (**E**) Venn diagram showing the suppression overlap of DEGs among QA4, QA5, DA4, and DA5 treatment groups, with numbers indicating gene counts and percentages within each category. (**F**) Quantitative analysis of gene CEACAM6 mRNA expression results, showing relative gene expression levels under different treatment conditions at a drug concentration of 10 μM. The data further confirm the transcriptional suppression induced by QA4 and QA5. Bars represent mean ± SD. (using a one-way ANOVA, *P < .05, **P < .01, ***P < .001, ****P < .0001.) (**G**) Schematic representation of the CEACAM6 promoter designed from the CEACAM6 promoter region (from −500 bp upstream to +10 bp downstream of the transcription start site). These sequences included the transcription factor SMAD3 binding site (CpApGpA) and the QA4/QA5 binding sites (CpG). A CD *in vitro* assay was performed to evaluate transcriptional suppression under QA4 and QA5 treatments.

Structural analyses revealed that QA compounds bind at the triplex junction and are associated with changes in DNA topology and local rigidity, raising the possibility that such binding may influence DNA-associated protein interactions [43]. To evaluate their effects on topoisomerase activity, we examined DNA relaxation mediated by TOP1 and TOP2, enzymes responsible for resolving DNA topological stress [44–47]. In topoisomerase assays performed without ethidium bromide, QA4 and QA5 caused a concentration-dependent persistence of supercoiled DNA, indicating impaired TOP1-mediated relaxation at concentrations above 10 μM (Fig. 5A). Under these assay conditions, the readout reflects DNA topology and relaxation efficiency rather than cleavage complex stabilization. Thus, the observed effect is consistent with suppression of productive TOP1-mediated relaxation rather than direct catalytic inhibition. By contrast, QA4 and QA5 did not affect TOP2-mediated DNA processing, as treatment did not alter the levels of nicked open or relaxed circular DNA (Fig. 5B), supporting selective modulation of TOP1-associated DNA relaxation. To identify the chemical elements contributing to TOP1 suppression, we analyzed related derivatives. The mono-intercalator 9-aminoacridine markedly reduced DNA relaxation, whereas the diquinoxaline-based bis-intercalators QU4 and QU5 showed no detectable effect (Supplementary Fig. S6B and C). The longer-linker derivative QA9 displayed attenuated suppression compared to QA4, highlighting the importance of linker geometry. Together, these findings indicate that the acridine chromophore and linker length critically influence DNA topology-dependent modulation of TOP1 activity.

Since the formation of triplex intermediates is coupled to both DNA replication and RNA transcription (Fig. 1A), to determine whether the structural effects induced by QA intercalation influence these processes, we first evaluated the impact of these compounds on DNA polymerase I activity. The results show that treatment with QA4 and QA5 showed no detectable inhibition of DNA polymerase I activity (Supplementary Fig. S7). Next, to explore the potential effects of QA compounds on transcriptional regulation, we performed RNA sequencing and DO enrichment analyses in QA-treated SW620 colorectal cancer cells. The transcriptome profiles revealed significant alterations enriched for colorectal cancer-related pathways (Fig. 5C and D). Consistently, QA4 and QA5 exhibited potent cytotoxicity in SW620 cells, with IC_50_ values of 7.2 ± 0.5 μM and 9.0 ± 0.1 μM, respectively, whereas higher IC_50_ values (∼10 μM) were observed in A549 lung cancer cells (Supplementary Fig. S8). To further examine gene expression changes associated with QA treatment, we performed a comparative Venn diagram analysis to identify both overlapping and unique patterns of DEGs following treatment with QA compounds (QA4 and QA5) and DA derivatives (DA4 and DA5). This analysis revealed 65 genes that were downregulated upon QA treatment but not by DA compounds (Fig. 5E), which may reflect transcriptional effects associated with the presence of the quinoxaline-containing scaffold. Among these, CEACAM6 (carcinoembryonic antigen-related cell adhesion molecule 6) exhibited the most pronounced downregulation in response to QA treatment. CEACAM6 is known to be overexpressed in ~70% of solid tumors, including gastrointestinal cancers, and acts as an oncoprotein that promotes cellular proliferation, migration, and invasion [48, 49]. This finding was further validated by RT-PCR, which demonstrated that CEACAM6 expression was suppressed by QA4, QA5, as well as QU4 and QU5, but not by DA4 or DA5. These results support an association between quinoxaline-containing compounds and repression of CEACAM6 expression (Fig. 5F). Notably, QA compounds exhibited stronger inhibitory effects than QU compounds, which may reflect contributions from both quinoxaline and acridine moieties. We further validated the transcriptional inhibition mediated by QA compounds by examining secondary structure changes in DNA sequences derived from the CEACAM6 promoter, specifically those containing transcription factor binding motifs (CpApGpA) adjacent to the quinoxaline intercalation site (CpG step). CD spectroscopy confirmed the binding of QA4 and QA5 to these promoter fragments, revealing ligand-induced conformational changes that suggest potential disruption of transcription factor–DNA interactions (Fig. 5G). Overall, these data show that QA compounds are associated with TOP1 suppression and transcriptional changes in cancer cells, with distinct contributions from the acridine and quinoxaline chromophores.

### QA compounds induce cell cycle redistribution and inhibit tumor growth in colorectal cancer xenograft models

**Figure 6.**
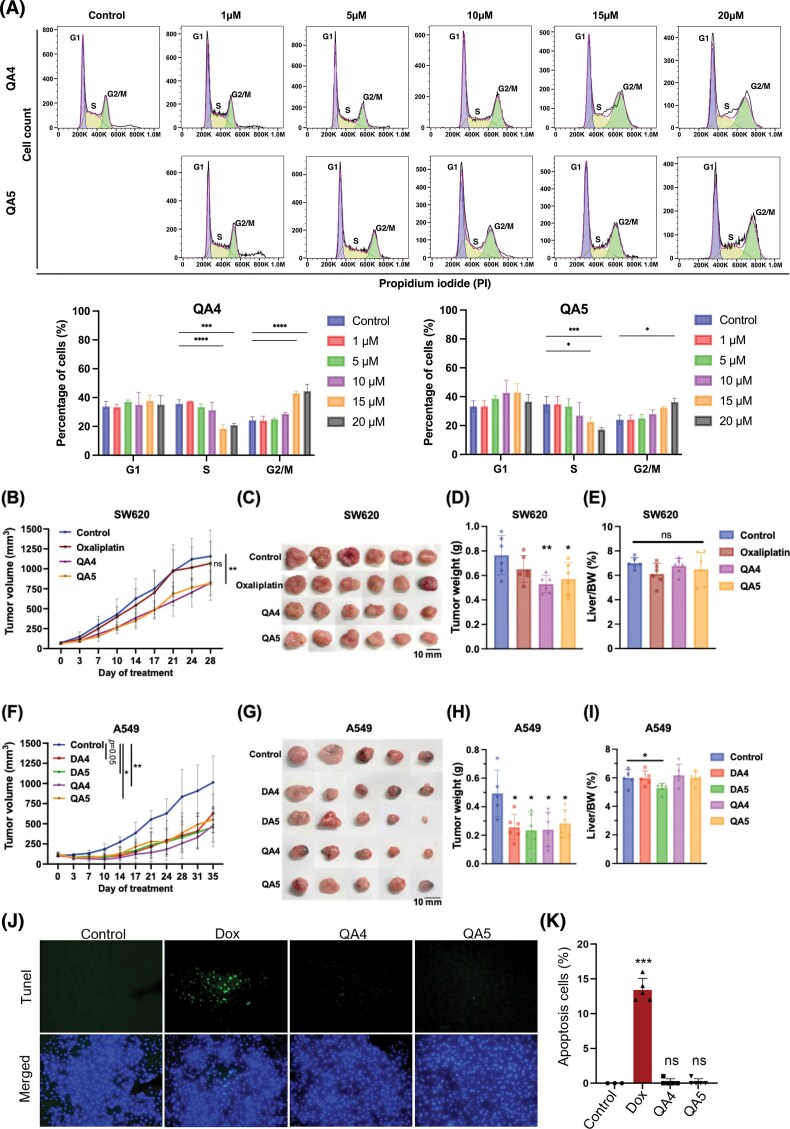
QA4 and QA5 induce cell cycle accumulation and inhibit tumor growth in colorectal (SW620) and lung (A549) cancer models. (**A**) Cell cycle analysis after 24 h treatment with various concentrations of QA4 and QA5. Control cells display a clear G1/S/G2–M distribution, serving as the reference for subsequent comparisons. (using a two-way ANOVA, *P < .05, **P < .01, ***P < .001, ****P < .0001.) (**B**) Therapeutic efficacy of oxaliplatin (1.5 mg/kg), QA4 (1.2 mg/kg), and QA5 (1.2 mg/kg) in SW620-bearing mice. The figure represents the SW620 tumor growth curve, images of the tumors with a scale bar of 10 mm (**C**), tumor weights (**D**), and the liver-to-body weight ratio after post-treatment at day 28 (**E**). (*n* = 6 per group) (**F**) A549 tumor bearing mice were received 1.2 mg/kg of DA4, DA5, QA4, and QA5 weekly for 5 weeks. The data represent the A549 tumor growth curve, pictures of tumors, scale bar 10 mm (**G**), tumor weights (**H**), and the liver-to-body weight ratio after post-treatment at day 35 (**I**). (*n* = 5–6 per group) Error bars are expressed as mean ± SD. Significance was compared with the vehicle group at the end of the study, using statistical analysis by an unpaired two-tailed Student’s *t*-test: *P < .05; **P < .01; ***P < .001; ns = not significant. (**J**) BALB/C mice were treated with control (5 mg/ml BSA) and high concentrations (6 mg/kg) of Dox, QA4, and QA5, and livers were harvested on day 4. The apoptotic cells were stained green fluorescence using the TUNEL assay on liver tissue sections. The image is shown at 400x magnification. (**K**) Percentages of apoptotic cells in liver tissues (control, *n* = 3; Dox, QA4, and QA5, *n* = 5). The data are shown as the means ± SD. Treatment groups were compared with the control group using a two-tailed Student’s *t*-test, ***P < .001; ns, not significant.

CEACAM6 has been reported to promote tumor proliferation through the regulation of cell cycle-associated factors such as cyclin D1 and CDK4 [49]. To examine whether QA treatment is associated with alterations in cell cycle progression, we analyzed cell cycle profiles of colorectal cancer cells following compound exposure. Using control cells with a well-defined G1/S/G2-M distribution, flow cytometry revealed a concentration-dependent redistribution of cells toward the G2/M phase, accompanied by a reduction in the S-phase population (Fig. 6A). These observations indicate that QA treatment is associated with cell cycle redistribution consistent with cellular stress responses, but do not alone define the underlying molecular mechanism [50, 51]. These observations are consistent with cell cycle redistribution patterns previously reported in contexts involving DNA topological stress. To further evaluate the antitumor activity of QA compounds *in vivo*, we assessed their efficacy in xenograft mouse models bearing SW620 colorectal tumors. QA treatment resulted in a significant reduction in tumor volume and weight compared with oxaliplatin, a clinically used chemotherapeutic agent (Fig. 6B–D). Comparable tumor growth inhibition was also observed in a lung cancer xenograft model, in which QA compounds, similar to DA compounds, markedly suppressed tumor progression (Fig. 6F–H). Treatment safety was evaluated by monitoring liver weight, histological analysis, and systemic parameters. Liver weights remained unchanged across treatment groups in both colorectal and lung cancer models (Fig. 6E and I). Consistent with this, TUNEL staining revealed no significant increase in apoptotic cells in liver tissues from QA4- or QA5-treated mice relative to control animals (Fig. 6J and K). In addition, no significant differences in body weight were observed throughout the treatment period (Supplementary Fig. S9), indicating minimal systemic toxicity. Collectively, these *in vivo* results demonstrate that QA compounds exhibit antitumor activity with a favorable safety profile in xenograft models. In addition, Annexin V/PI staining revealed that QA4 and QA5 induce apoptotic cell death in SW620 cells, providing further cellular-level support for their anticancer activity (Supplementary Fig. S10).

## Discussion

As the leading cause of death worldwide, cancer highlights the urgent need for improved chemotherapeutic agents [4, 52–55]. DNA intercalators constitute a prominent class of chemotherapeutic drugs, yet their clinical utility is hampered by non-specific cytotoxicity resulting from broad genomic binding [56]. Conventional DNA intercalators face a fundamental therapeutic limitation: their sequence-based targeting leads to genome-wide interactions and indiscriminate cytotoxicity. Mono-intercalators such as actinomycin D and DOX insert into CpG-rich regions throughout the genome without structural selectivity, while symmetric bis-intercalators such as echinomycin, XR5944, and ditercalinium achieve enhanced affinity but remain confined to ubiquitous sequence motifs [57–62]. However, their specificity remains confined to common sequence motifs (e.g. GpC or CpG), which are widely distributed across the genome, thereby limiting their therapeutic potential. Recent structural studies demonstrating that certain bis-intercalators can engage two DNA duplexes simultaneously revealed that higher-order DNA structures may serve as conformationally selective targets [11, 63]. This conceptual advance shifts the paradigm from sequence-dependent to topology-driven recognition. Based on this rationale, we hypothesized that asymmetric coupling of chromophores with distinct intercalation strengths and complementary biological functions could achieve structural preference for topologically perturbed DNA architectures together with dual-pathway interference. Structurally, the dual chromophores operate within a conformationally constrained junctional environment, where their insertion is coordinated by local DNA distortion rather than by two independent classical duplex intercalation events. Supporting this concept, studies on unsymmetrical acridine derivatives (UAs), composed of imidazoacridinone and 1-nitroacridine connected by an aminoalkyl linker, have shown that asymmetrical linkage of distinct acridine units gives rise to emergent chemical and biological properties that exceed the simple additive effects of each moiety [64]. These findings imply that asymmetrically designed compounds may exhibit unique chemical features, allowing each moiety to retain its function while acting synergistically to enhance biological activity. Additionally, the intercalation strength of DNA-binding moieties has been reported to correlate directly with both therapeutic efficacy and off-target cytotoxicity. Accordingly, combining strong and weak intercalating units within a single molecule may represent a rational strategy to optimize the therapeutic index by preserving antitumor activity while reducing undesired cellular toxicity. This design concept integrates complementary features that balance potency with selectivity. The combination of a strong and a mild intercalating unit may enhance therapeutic efficacy while potentially minimizing nonspecific cytotoxicity.

Although QA compounds are designed as bis-intercalators and are therefore expected to associate broadly with canonical duplex DNA (Supplementary Fig. S11), duplex binding alone is unlikely to fully account for the observed biological effects. Acridine-based chromophores are known to interact with nucleic acids in diverse structural contexts; however, the present framework focuses on DNA structural modulation under conditions of topological stress. Transcription- and replication-associated torsional stress can transiently generate non-canonical DNA architectures, including triplex and junctional structures [25]. Such conformations have been reported to modulate TOP1 activity in a position- and orientation-dependent manner [26]. Our crystallographic analyses indicate that QA compounds rearrange a junctional DNA architecture, suggesting that ligand-induced structural perturbation may influence TOP1-mediated relaxation efficiency. In this context, duplex DNA likely represents a general binding substrate, whereas structurally distorted junctional regions may constitute conformationally sensitive sites where ligand-induced perturbation is enhanced. To evaluate whether QA compounds indiscriminately perturb other non-canonical DNA conformations, we performed additional CD analyses using a canonical duplex control sequence, the triplex junction-forming sequence examined in this study, a human telomeric i-motif, and a human telomeric G-quadruplex (hybrid form) (Supplementary Fig. S12). Under identical conditions, QA compounds induced markedly greater conformational perturbation in the triplex junction-forming architecture than in the canonical duplex, i-motif, or G-quadruplex. These observations suggest that QA compounds exhibit a structural preference for triplex junction architectures compared with perfect duplex DNA, rather than indiscriminately disrupting diverse non-B-DNA structures. Structural analysis further showed that acridine and quinoxaline moieties exhibit distinct stacking geometries and intercalation depths at CpG steps, with acridine generating denser π-stacking than quinoxaline. This difference explains the complementary biological roles of the two chromophores: acridine produces local helical distortion that impedes TOP1 access, while quinoxaline perturbs the groove environment and attenuates transcription factor binding. Consistent with this division of labor, QA compounds show reduced toxicity relative to the strong/strong diacridine analog DA and gain TOP1 inhibitory activity and stronger transcriptional repression compared with the weak/weak quinoxaline analog QU. Replacing dual acridine with acridine plus quinoxaline abolishes TOP2 inhibition but increases specificity for TOP1, which likely reflects distinct structural requirements of TOP1 and TOP2, and the ability of diacridines to stabilize TOP2 cleavage complexes by bridging two intercalation sites [11]. Importantly, although QA and DA compounds produce partially overlapping cellular outcomes such as apoptosis and tumor growth inhibition, their mechanistic bases diverge: DA compounds act primarily through repressing TOP2-mediated relaxation and DNA damage accumulation, whereas QA compounds preferentially target stressed junctional DNA, suppress TOP1 activity, and modulate transcriptional programs.

These structural effects occur in a physiologically relevant context. Polyamines such as spermine stabilize triplex and junctional DNA at physiological ionic strength by neutralizing phosphate repulsion and bridging adjacent helices [30, 31]. This intrinsic stabilizing effect suggests that triplex junctions may transiently form *in vivo* under torsional stress, providing a physiological context for the observed interactions of QA compounds. In addition to this global electrostatic contribution, our crystallographic structures indicate that polyamines can also adopt spatially defined positions near groove and junctional regions, where they form localized contacts with the distorted DNA backbone. Such interactions likely complement electrostatic screening by stabilizing specific junction geometries once formed, thereby fine-tuning the conformational landscape of the triplex junction architecture. Recent studies have further established DNA junctions as distinct non-B-DNA targets with well-defined ligand-binding cavities amenable to selective recognition by small molecules [33, 65]. In particular, azacryptand-based ligands have been shown to stabilize three-way DNA junctions in human cells, triggering γH2AX-marked DNA damage and synthetic lethality when combined with DNA repair inhibitors. A related study further demonstrated that azacryptand derivatives can concurrently recognize multiple higher-order DNA structures, underscoring the feasibility of dual-targeting strategies [32]. These findings highlight the therapeutic potential of targeting junctional DNA structures, consistent with our observation that QA compounds disrupt triplex junctions and suppress TOP1-mediated DNA relaxation through modulation of DNA structure. To evaluate the specific structural impact of incorporating quinoxaline into asymmetric bis-intercalators, we compared the QA–DNA complex structures with previously reported symmetric bis-acridine intercalators (DA compounds) bound to the d(CGTATACG)_2_ duplex (PDB IDs: 8WQ7 and 8W7W) [11]. The comparison revealed that QA compounds induce a more pronounced increase in roll angles within the DNA duplex relative to acridine-only structures. This effect is likely due to the quinoxaline group, which may confer additional functional properties beyond topological distortion, such as disrupting transcription factor–DNA interactions. Specifically, SMAD3 is a transcription factor essential for activating CEACAM6 expression through recognition of the canonical CAGA sequence. It preferentially binds to B-form DNA, which presents a well-defined major groove (~11 Å wide) that accommodates its conserved β-hairpin domain, enabling stable and sequence-specific interactions [66–69]. Therefore, ligand-induced DNA bending and groove deformation toward an A-DNA-like geometry may disrupt the optimal binding conformation required for SMAD3 [70, 71], ultimately leading to diminished transcriptional activation of CEACAM6. Functional studies revealed that quinoxaline and acridine individually suppress CEACAM6 and TOP1, respectively, whereas QA compounds target both, showing additive effects on CEACAM6 repression. Consistent with structural analyses, this suggests a synergistic effect arising from triplex dissociation and quinoxaline-induced A-like DNA conformation. Finally, linker length modulates both binding and activity: QA4, with a four-carbon linker, shows stronger stacking, greater structural distortion, and improved biological efficacy than the five-carbon analog QA5. QA4, which contains a four-carbon linker, exhibited enhanced stacking interactions with DNA, correlating with greater structural distortion and improved biological efficacy compared to the five-carbon analog QA5. In contrast, previous studies on symmetric bi-acridine bis-intercalators reported that five-carbon linkers, relative to four-carbon ones, were associated with increased inter-duplex contacts and enhanced anticancer activity. This structure–activity relationship highlights the critical role of linker properties in bi-targeting DNA intercalator design and serves as a reference for developing structurally optimized bis-intercalators.

Quinoxaline is delineated here as an intercalating unit with distinct structural and functional roles within asymmetric bis-intercalators. Its transcriptional inhibitory potential was initially demonstrated in the symmetric bis-intercalator ECHI, which inserts two quinoxaline groups at CpG-flanked sites. ECHI induces substantial DNA backbone distortion, thought to interfere with transcription factor binding, such as HIF-1 at promoter regions [60]. The structural consequences of ECHI have been addressed by high-resolution crystal structures. For instance, in PDB entry 5YTY, two ECHI molecules bind to a DNA duplex flanked by one base pair, with four quinoxaline moieties intercalated at GpT/ApC and GpA/TpC steps [72]. This binding induces a modest decrease in roll (approximately −2.1°) and a pronounced reduction in twist angle (~14.2°). Conversely, in PDB entry 1XVK, two ECHI molecules bind to a DNA duplex flanked by two base pairs, where quinoxaline intercalates at GpT/ApC and GpC/GpC steps [73], leading to a substantial increase in roll angle (~40.7°) and a reduction in twist angle (~35.3°), relative to canonical B-DNA values (0° for roll and 36° for twist) (Supplementary Fig. S13A). These findings indicate that the structural impact of quinoxaline is sensitive to intercalation context, including flanking sequence composition. Despite these insights, the structural impact of a single quinoxaline moiety within an asymmetric bis-intercalator context had not been previously characterized. By comparing QA–DNA complexes with diacridine bis-intercalator–DNA structures exhibiting similar intercalation patterns (Supplementary Fig. S13B), we found that QA compounds induce a pronounced increase in roll angle within the intercalated duplex that is notably absent in acridine-only analogs [11]. This observation highlights the unique contribution of quinoxaline to DNA distortion.

In conclusion, this study demonstrates that asymmetric bis-intercalators, incorporating both acridine and quinoxaline, achieve a balanced interplay between therapeutic efficacy and safety by targeting DNA triplex junction-associated architectures. The synergistic combination of these moieties enhances antitumor activity while minimizing toxicity, representing a promising strategy for the rational design of next-generation DNA-targeting therapeutics.

## Supplementary Material

gkag520_Supplemental_File

## Data Availability

The atomic coordinates and structure factors for the reported crystal structures have been deposited in the Protein Data Bank under the accession Nos 9V87 (https://doi.org/10.2210/pdb9b87/pdb), 9V88 (https://doi.org/10.2210/pdb9v88/pdb), and 9V89 (https://doi.org/10.2210/pdb9v89/pdb) for ligand-free DNA, QA4–DNA, and QA5–DNA complex structures, respectively.
