# Supplementary material for: Structural basis for asymmetric bis-intercalator targeting of DNA triplex junctions enabling dual inhibition of topoisomerase I and oncogene transcription
Source: Nucleic Acids Res. 2026 May 28;54(10):gkag520. doi: 10.1093/nar/gkag520 (PMC13216744; doi:10.1093/nar/gkag520)
Supplement: gkag520_Supplemental_File [file gkag520_supplemental_file.docx]

**Supplementary Information**

**Structural basis for asymmetric bis-intercalator targeting of DNA triplex junctions enabling dual inhibition of topoisomerase I and oncogene transcription**

Shun-Ching Wang^1,2^†, Chang-Chih Hsieh^4^†, Tzu-Chun Yuan^2^, Shan-Meng Lin^2^, Chia-Wei Chen^3^, Chih-Chun Chang^5^, Shih-Chun Huang^1,2^, En-Chi Wang^3^, Yu-Jhen Huang^3^, Ming-Hsi Chiang^4^, Yih-Chern Horng^3^*, and Ming-Hon Hou^1,2,5,6^*

*Corresponding author(s): Tel: +886 4 2284 0338 (Ext 7011); Fax: +886 4 2285 9329; Email: mhho@nchu.edu.tw and ychorng@cc.ncue.edu.tw

**This PDF file includes:**

Supplementary Note

Supplementary Figure S1 to S13

Supplementary Table S1 to S3

Supplementary References


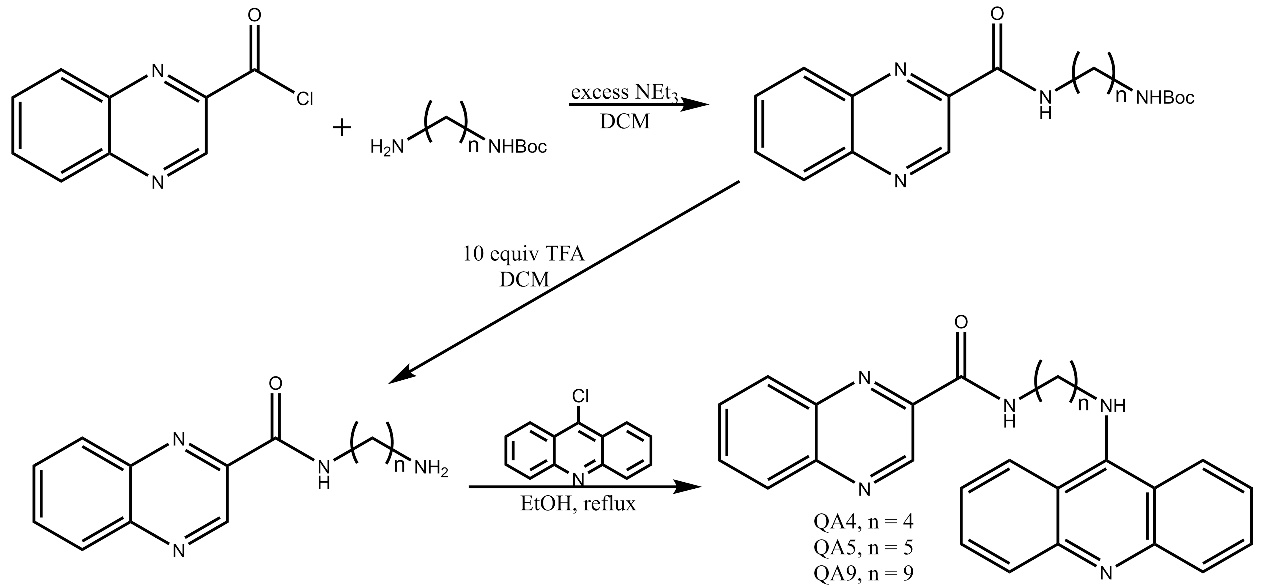
**Supplementary Note**


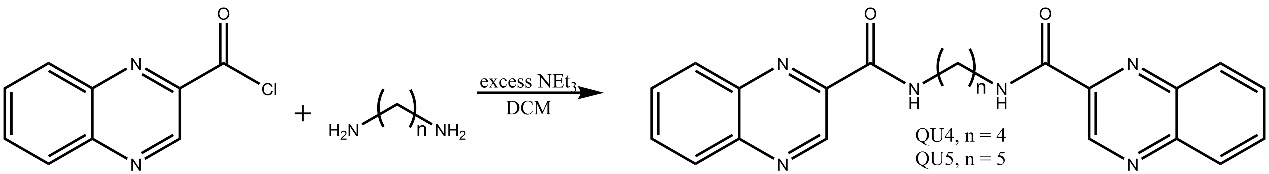
**Scheme S1. The synthesis of QA compounds.**

**Scheme S2. The synthesis of QU compounds.**

**Experimental**

**General procedures**

Commercially available chemicals, including 9-chloroacridine, 2-quinoxaloyl chloride, *t*-butyl *N*-(4-aminobutyl) carbamate (*N*-Boc-1,4-diaminobutane), and *t*-butyl *N*-(5-aminopentyl) carbamate (*N*-Boc-1,5-diaminopentane), were purchased from Sigma-Aldrich or TCI, and used as received. All reagents were of analytical grade and used without further purification. Ethanol (EtOH) was distilled under nitrogen using CaH_2_ as a drying reagent and stored in dried N_2_-filled reservoirs containing 4 Å molecular sieves before use. ^1^H NMR spectra were collected on a Bruker Avance 300 spectrometer. Chemical shifts for ^1^H spectra were recorded in ppm relative to the residual proton (^1^H of CDCl_3_: *δ* 7.24 and ^1^H of DMSO-*d*_6_: *δ* 2.50). DFT calculations were performed on the Gaussian 09 program. Geometry optimizations were conducted using the B3LYP functional and 6-31G* basis sets. The solvation free-energy was investigated using the self-consistent reaction field (SCRF) and solvation model (SMD) ([1](#_ENREF_1)). Elemental analyses and MS spectrometry were performed on a Heraeus CHN-OS Rapid Elemental Analyzer and JEOL JMX-SX/SX 102A Mass Spectrometer at the Instruments Center of National Chung Hsing University, Taiwan.

**Synthesis of QA compounds**

A solution of 2-quinoxaloyl chloride (1 equiv, 1 mmol) in 15 mL of dichloromethane (DCM) was slowly added over 20 min to a solution of N-Boc-diaminoalkane (1 equiv) and triethylamine (NEt₃, 6 mL) in 30 mL of DCM. The resulting mixture was stirred overnight at room temperature. Subsequently, the reaction mixture was washed with water (100 mL × 5). The organic layer was collected, dried over anhydrous MgSO₄, filtered, and concentrated under reduced pressure to afford the Boc-protected products.

tert-butyl (4-(quinoxaline-2-carboxamido) butyl) carbamate (96% yield). 1H NMR (CDCl3): δ 9.65 (s, 1H), 8.17 (m, 1H), 8.10 (m, 1H), 8.04 (br, 1H), 7.84 (m, 2H), 4.62 (br, 1H), 3.55 (q, 2H), 3.18 (q, 2H), 1.71 (m, 2H), 1.65 (s, 9H), 1.60 (m, 2H). Elem Anal. Calcd (%) for C18H24N4O3: C 62.77; H 7.02; N 16.27. Found: C 62.55; H 6.89; N 16.51.

tert-butyl (5-(quinoxaline-2-carboxamido) pentyl) carbamate (97% yield). 1H NMR (CDCl3): δ 9.66 (s, 1H), 8.17 (m, 1H), 8.11 (m, 1H), 8.01 (br, 1H), 7.84 (m, 2H), 4.56 (br, 1H), 3.54 (q, 2H), 3.12 (q, 2H), 1.71 (qu, 2H), 1.60 (s, 9H), 1.55 (m, 2H), 1.44 (m, 2H). Elem Anal. Calcd (%) for C19H26N4O3: C 63.67; H 7.31; N 15.63. Found: C 63.90; H 7.44; N 15.48.

tert-butyl (9-(quinoxaline-2-carboxamido) nonyl) carbamate (81% yield). 1H NMR (CDCl3): δ 9.65 (s, 1H), 8.15 (m, 1H), 8.08 (m, 1H), 7.98 (br, 1H), 7.82 (m, 2H), 4.51 (br, 1H), 3.51 (q, 2H), 3.06 (q, 2H), 1.66 (qu, 2H), 1.40 (m, 15H), 1.34 (m, 2H), 1.27 (m, 4H). Elem Anal. Calcd (%) for C23H34N4O3: C 66.64; H 8.27; N 13.52. Found: C 66.87; H 8.14; N 13.31.

Ten equivalents of trifluoroacetic acid (TFA) were added to a solution of the Boc-protected compound (1.0 equiv) in 30 mL of dichloromethane (DCM), and the resulting mixture was stirred for 1 h. A saturated aqueous solution of NaHCO₃ (100 mL) was then slowly added to quench the reaction until gas evolution ceased. The aqueous layer was concentrated until a large amount of solid precipitated. The resulting mixture was extracted with DCM (20 mL × 3), and the combined organic layers were dried over anhydrous MgSO₄. After filtration, the solvent was removed under reduced pressure to afford the amino product.

N-(4-aminobutyl)-2-quinoxaline carboxamide (65% yield). 1H NMR (CDCl3): δ 9.66 (s, 1H), 8.17 (m, 1H), 8.13 (br, 1H), 8.09 (m, 1H), 7.84 (m, 2H), 3.59 (q, 2H), 2.76 (t, 2H), 1.74 (qu, 2H), 1.59 (qu, 2H). Elem Anal. Calcd (%) for C13H16N4O: C 63.91; H 6.60; N 22.93. Found: C 63.88; H 6.23; N 22.72.

N-(5-aminopentyl)-2-quinoxaline carboxamide (62% yield).1H NMR (CDCl3): δ 9.66 (s, 1H), 8.17 (m, 1H), 8.10 (m, 1H), 8.02 (br, 1H), 7.84 (m, 2H), 3.54 (q, 2H), 2.71 (t, 2H), 1.71 (qu, 2H), 1.61, (m, 2H), 1.49 (m, 2H). Elem Anal. Calcd (%) for C14H18N4O: C 65.09; H 7.02; N 21.69. Found: C 65.38; H 7.36; N 21.60.

N-(9-aminononyl)-2-quinoxaline carboxamide (59% yield).1H NMR (CDCl3): δ 9.66 (s, 1H), 8.17 (m, 1H), 8.10 (m, 1H), 8.02 (br, 1H), 7.84 (m, 2H), 3.54 (q, 2H), 2.71 (t, 2H), 1.71 (qu, 2H), 1.61, (m, 2H), 1.49 (m, 2H). Elem Anal. Calcd (%) for C18H26N4O: C 68.76; H 8.34; N 17.82. Found: C 68.92; H 8.36; N 17.60.

The amino products (1 equiv) and 9-chloroacridine (1 equiv) were dissolved in ethanol (EtOH, 30 mL), and the solution was refluxed at 90 ºC overnight. After completion of the reaction, the mixture was cooled to room temperature and concentrated under reduced pressure. The residue was washed with diethyl ether (50 mL) to induce precipitation. The resulting precipitate was collected by filtration and dried under vacuum to afford the final product.

**QA4** (92% yield). ^1^H NMR (DMSO-*d6*): *δ* 9.41 (s, 2H), 9.13 (t, 1H), 8.60 (d, 2H), 8.16 (m, 2H), 7.98 (m, 2H), 7.91 (d, 4H), 7.49 (qu, 2H), 4.15 (t, 2H), 3.44 (q, 2H), 1.99 (qu, 2H), 1.74 (qu, 2H). Elem Anal. Calcd (%) for C26H23N5O: C 74.09; H 5.50; N 16.62. Found: C 73.87; H 5.17; N 17.01. HRMS Calcd for C26H24N5O [M+H] ^+^: m/z 422.1975. Found: 422.1968.

**QA5** (70% yield). ^1^H NMR (DMSO-*d6*): *δ* 9.77 (t, 1H), 9.39 (s, 1H), 9.08 (t, 1H), 8.54 (d, 2H), 8.17 (m, 2H), 7.98 (m, 2H), 7.91 (d, 2H), 7.84 (d, 2H), 7.49 (t, 2H), 4.09 (q, 2H), 3.57 (q, 2H), 1.96 (qu, 2H), 1.65 (qu, 2H), 1.46 (qu, 2H). Elem Anal. Calcd (%) for C27H25N5O: C 74.46; H 5.79; N 16.08. Found: C 74.83; H 5.80; N 15.84. HRMS Calcd for C27H26N5O [M+H] ^+^: m/z 436.2132. Found: 436.2127.

(t, 2H), 1.71 (qu, 2H), 1.61, (m, 2H), 1.49 (m, 2H). Elem Anal. Calcd (%) for C14H18N4O: C 65.09; H 7.02; N 21.69. Found: C 65.38; H 7.36; N 21.60.

**QA9** (22% yield). ^1^H NMR (DMSO-*d6*): *δ* 9.45 (s, 1H), 9.07 (t, 1H), 8.54 (m, 2H), 8.17 (m, 2H), 7.97 (t, 4H), 7.87 (d, 2H), 7.54 (t, 2H), 4.06 (t, 2H), 3.47 (q, 2H), 1.88 (qu, 2H), 1.57 (qu, 2H), 1.28 (m, 10H). Elem Anal. Calcd (%) for C31H33N5O: C 75.73; H 6.77; N 14.25. Found: C 75.88; H 6.53; N 14.34. HRMS Calcd for C31H34N5O [M+H] ^+^: m/z 492.2758. Found: 492.2768.

**Synthesis of QU compounds**

Diaminoalkane (0.5 equiv, 0.5 mmol) was dissolved in DCM (20 mL) to afford a clear and colorless solution in a 100 mL single-neck round-bottom flask. In a separate 100 mL flask, 2-quinoxalinoyl chloride (1.0 equiv) was dissolved in DCM (15 mL), giving a deep purple solution. This solution was then added dropwise to the diaminoalkane solution over 10 min under stirring using a dropping funnel. During the addition, the reaction mixture immediately changed color from colorless to deep purple. The mixture was stirred at room temperature overnight. Upon completion of the reaction, the solution color gradually changed from deep purple to purple-red.

The purple-red solution was transferred to an Erlenmeyer flask and washed with water (100 mL) two to four times. During the washing process, the aqueous layer color changed from clear orange to pale. The organic layer was then separated and dried over anhydrous MgSO₄. After filtration to remove the drying agent, the solvent was removed under reduced pressure to afford the product as an orange-yellow solid.

**QU4** (79% yield). ^1^H NMR (CDCl_3_): *δ* 9.61 (s, 2H), 8.10 (m, 4H), 8.03 (d, 2H), 7.78 (m, 4H), 3.59 (m, 4H), 1.8 (m, 4H). ^13^C{^1^H} NMR (CDCl_3_): *δ* 163.37, 143.77, 143.33, 140.13, 131.54, 130.81, 129.53, 129.41, 39.17, 27.25. Elem Anal. Calcd (%) for C22H20N6O2: C 65.99; H 5.03; N 20.99. Found: C 65.83; H 5.22; N 20.79. HRMS Calcd for C22H21N6O2 [M+H] ^+^: m/z 401.1721. Found: 401.1714.

**QU5** (75% yield). ^1^H NMR (CDCl_3_): *δ* 9.59 (s, 2H), 8.09 (d, 4H), 7.99 (d, 4H), 7.76 (m, 4H), 3.53 (q, 4H), 1.74 (m, 4H), 1.53 (m, 2H). ^13^C{^1^H} NMR (CDCl_3_): *δ* 163.30, 143.82, 143.41, 140.15, 131.50, 130.78, 129.52, 129.44, 39.31, 29.34, 24.27. Elem Anal. Calcd (%) for C23H22N6O2: C 66.65; H 5.35; N 20.28. Found: C 66.59; H 5.17; N 20.37. HRMS Calcd for C23H23N6O2 [M+H] ^+^: m/z 429.2034. Found: 429.2022.

**Supplementary Figures**


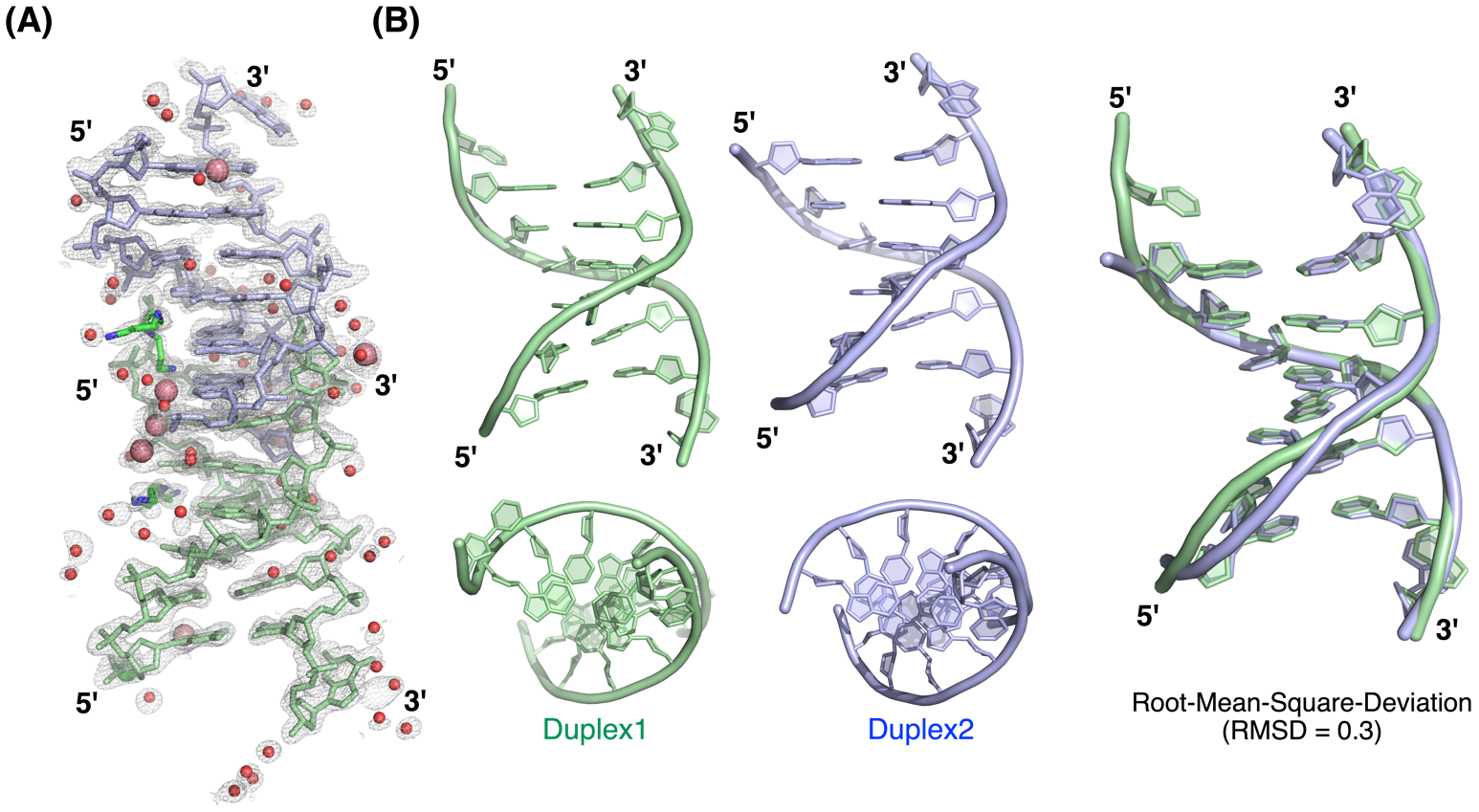


**Figure S1. Crystal structure of d(CGTTAACG)_2_ DNA only. (A)** 2F*o*-F*c* electron density maps of the refined structures of the d(CGTTAACG)_2_ in an asymmetric unit, contoured at 1.0 sigma. **(B)** Comparison of two neighboring duplexes in crystal symmetry shows identical duplexes seen from the side and from above. The superimposition of these duplexes shows a root mean square deviation of 0.3 Å.


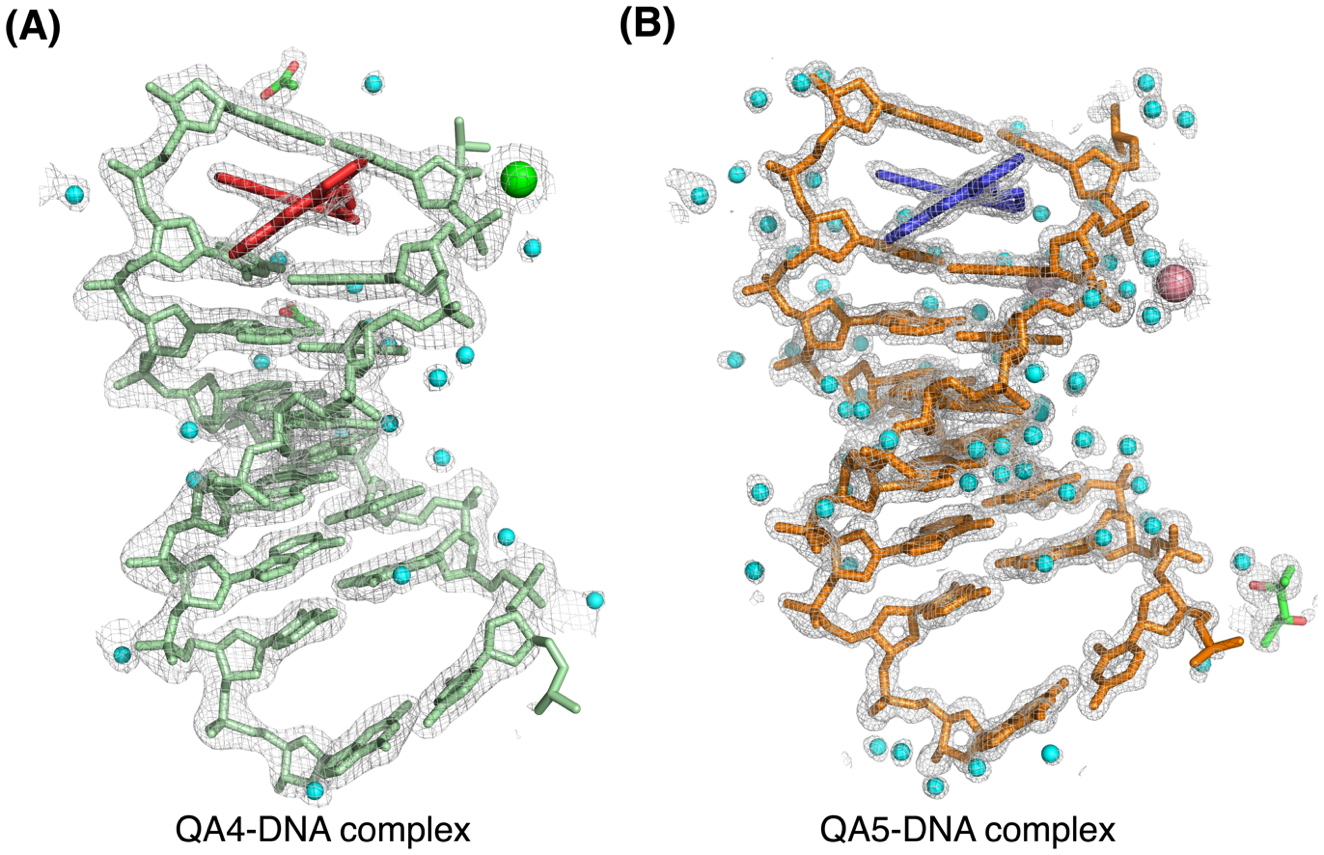


**Figure S2. 2F*o*-F*c* electron density maps of the refined structures contoured at 1.0 sigma.** In the depicted figures, **(A)** QA4-DNA complex and **(B)** QA5-DNA complex. DNA backbones are represented in pale green and orange, while compounds are shown in red and blue stick representation. Ions, including calcium (green), cobalt (light pink), and water (cyan), are shown in spheres. The increased separation at terminal base-pair steps corresponds to ligand-induced helical rise expansion consistent with the refined structural parameters.


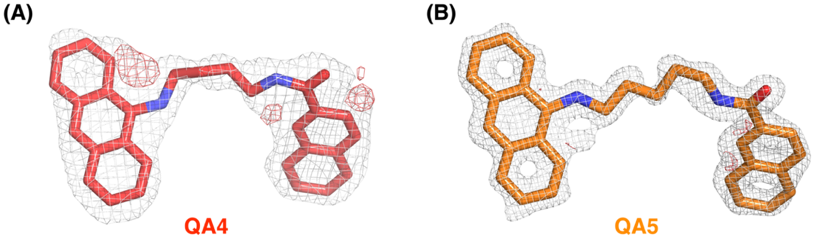


**Figure S3. Association between the electron density map quality and the atomic models of QA4 and QA5 in two distinct crystal structures.** The refined 2mF*o*-DF*c* maximum likelihood-weighted Fourier electron density map in xplor format is shown, contoured at the 1.0 sigma level with a 2.0 Å carve radius (grey mesh). The mF*o*-DF*c* difference map (red mesh at 3.0 sigma) shows a good fit of the geometries of **(A)** QA4 (red sticks) and **(B)** QA5 (orange sticks) geometries in both crystal structures.


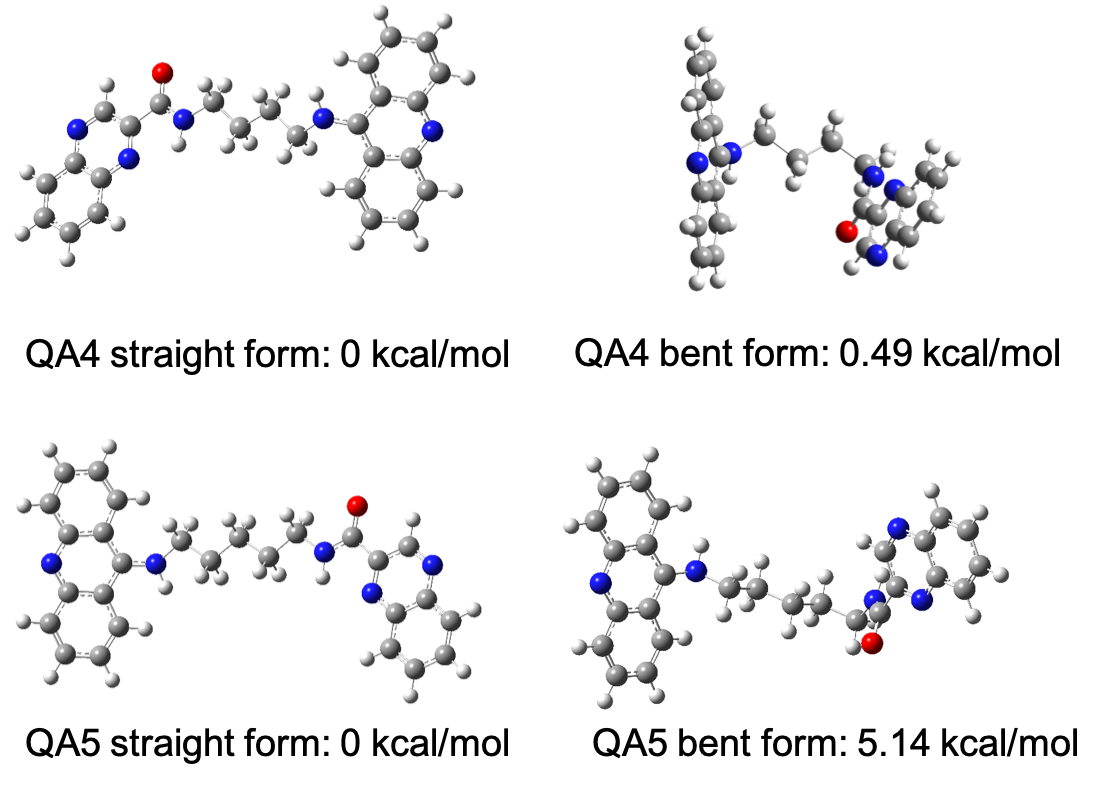


**Figure S4. Optimized molecular structures of QA4 and QA5 determined by density functional theory (DFT) calculations.** Carbon atoms are depicted in gray, nitrogen in blue, oxygen in red, and hydrogen in white.


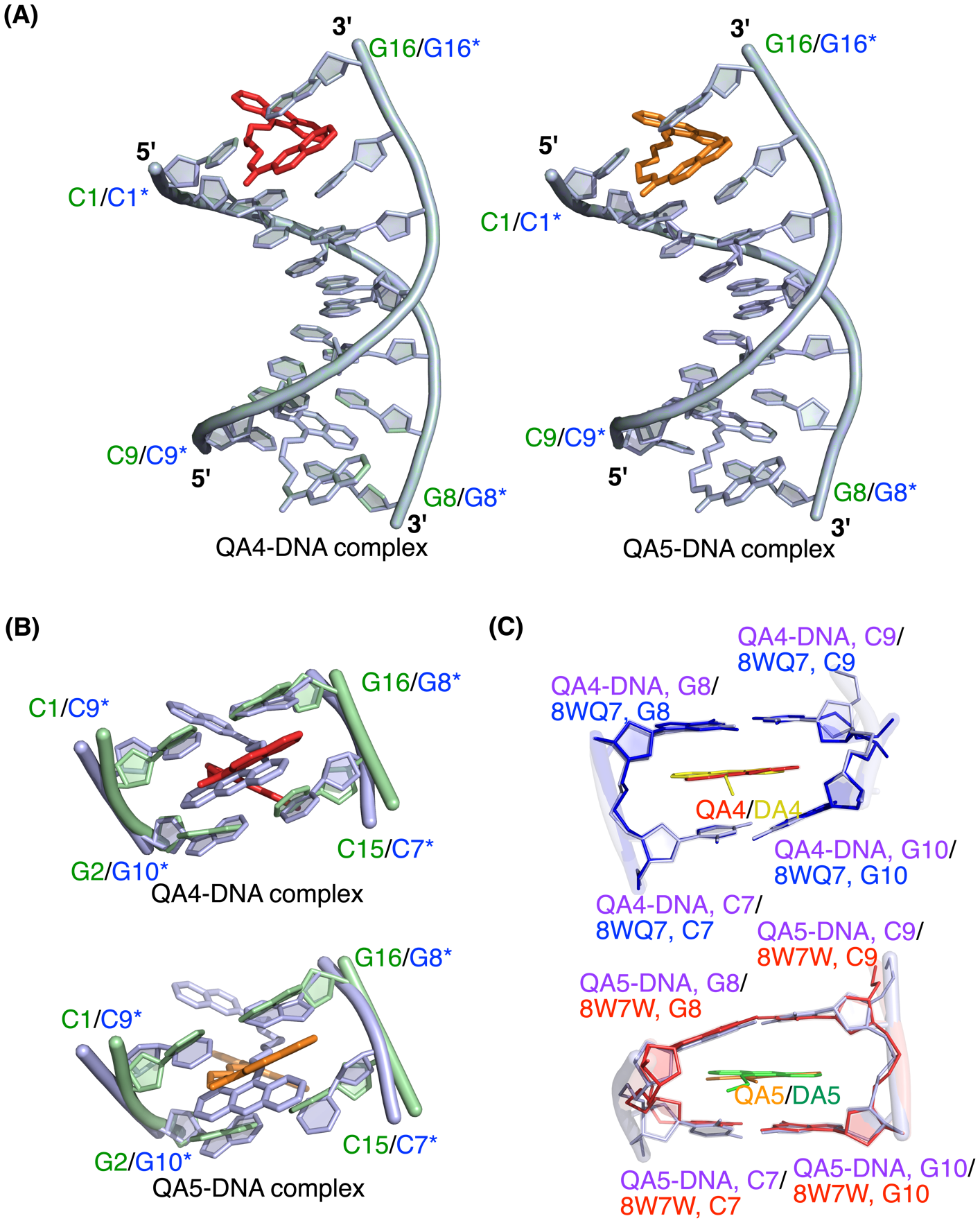


**Figure S5. Superimposition of QA4-DNA and QA5-DNA complex structures.** **(A)** Two compound-induced bis-intercalation DNA duplexes, represented by pale green and light blue colored cartoons, are overlaid in the QA4-DNA complex (left panel) and QA5-DNA complex (right panel). This comparison illustrates the similarities between these duplexes. **(B)** The upper chromophore binding site, specifically C1: G16/G2: C15 of one duplex (pale green colored cartoons), is superimposed with the corresponding binding site, C9*: G8*/G10*: C7*, of another duplex (light blue colored cartoons). This comparison highlights the differences in bis-intercalation sites between the QA4 and QA5 structures. **(C)** Structural superimposition of the acridine moieties (shown in sticks) intercalated at the C7pG8/C9pG10 site in QA4- and QA5-bound DNA (light blue and blue, respectively) with the corresponding site in previously reported DA4- and DA5-bound DNA (light blue and red, respectively).


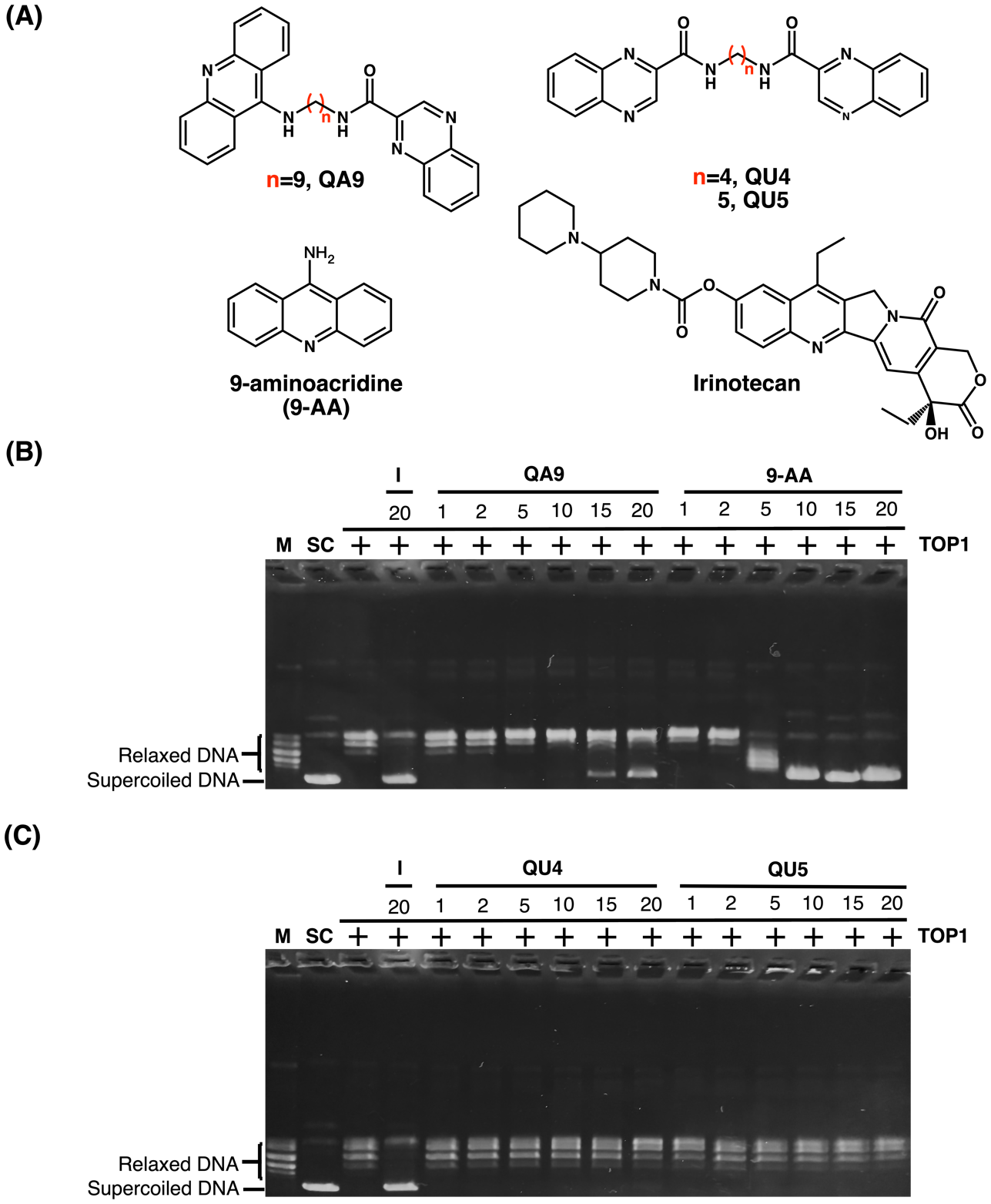


**Figure S6. Topoisomerase I (TOP1) activity assay (A)** Chemical structures of tested compounds used for topoisomerase I (TOP1) activity assay**.** QA9**:** An asymmetric bis-intercalator characterized by a long hydrocarbon linker chain, QU4 and 5: Diquinoxaline bis-intercalators connected by hydrocarbon linker chains, 9-AA: A mono-intercalating precursor to the aforementioned intercalators. **(B)**, and **(C)** representative gel for the topoisomerase I (TOP1) activity assay. Lane 1: Marker; Lane 2: Supercoiled DNA (pHOT1 plasmid); Lane 3: pHOT1 DNA with TOP1; Lane 4: pHOT1 DNA with TOP1 and 20 µM irinotecan; Lanes 5-10: Supercoiled DNA with TOP1 and various compounds at 1, 2, 5, 10, 15, and 20 µM, respectively; Lanes 11-16: Supercoiled DNA with TOP1 and a different set of compounds at the same concentrations (1, 2, 5, 10, 15, and 20 µM, respectively).


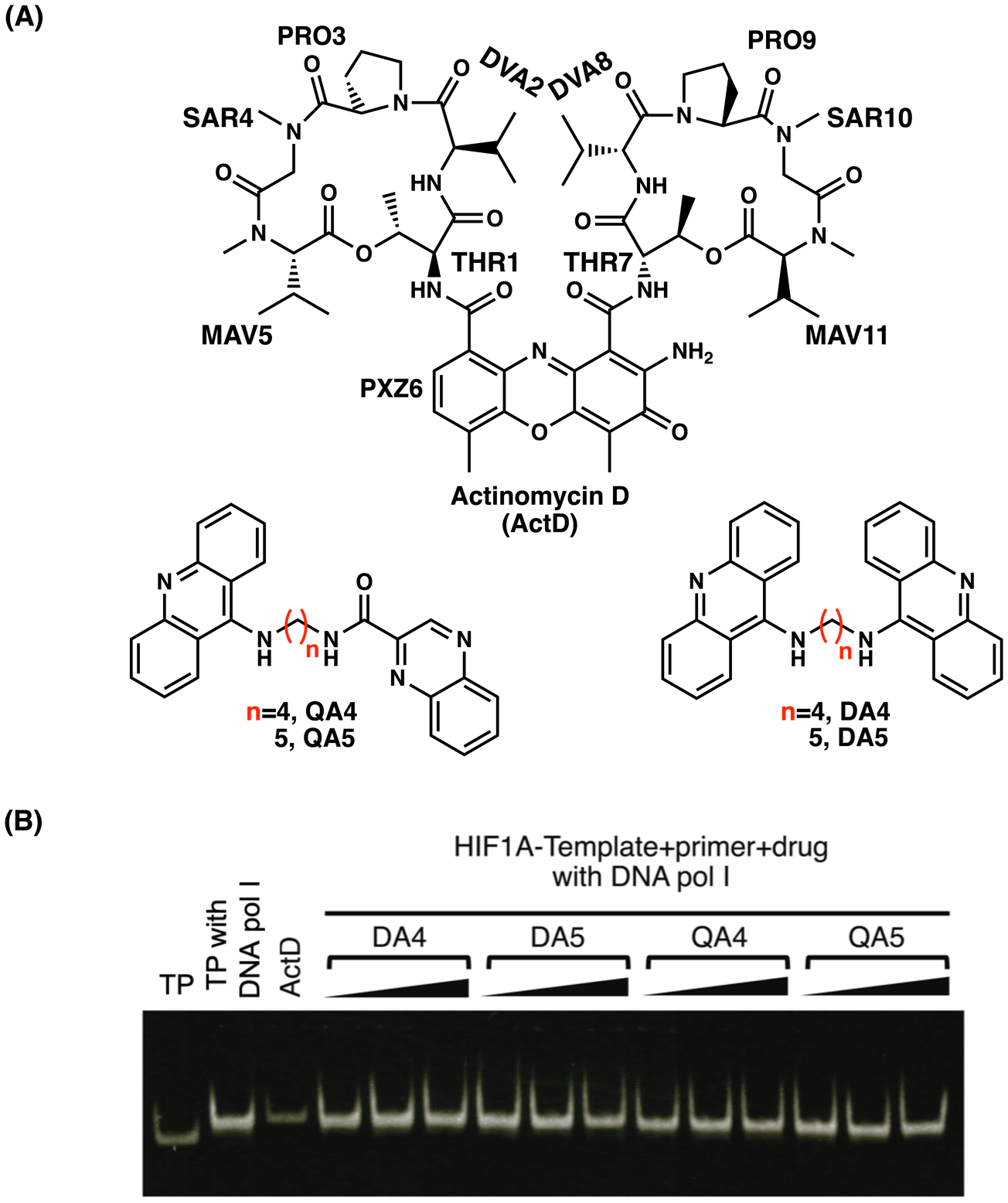


**Figure S7. The representative gel for the DNA polymerase I activity assay.** **(A)** HIF1A, Hypoxia-inducible factor 1-alpha; DNA pol I, DNA polymerase I; DA, Diacridine intercalator; QA, quinoxaline-acridine asymmetric intercalator; ActD, Actinomycin D. **(B)** Template containing the HIF1A promoter sequences. Lane 1, The control contained the HIF1A template and primer only; Lane 2, HIF1A template and primer with DNA polymerase I; Lane 3, the positive control contained the HIF1A template and primer with DNA polymerase I incubated with 25 µM actinomycin D (ActD); Lane 4-6, HIF1A template and primer with DA4 10, 25, 50 µM and was incubated with DNA polymerase I; Lane 7-9, HIF1A template and primer with DA5 10, 25, 50 µM and was incubated with DNA polymerase I; Lane 10-12, HIF1A template and primer with QA4 10, 25, 50 µM and was incubated with DNA polymerase I; Lane 13-15, HIF1A template and primer with QA5 10, 25, 50 µM and was incubated with DNA polymerase I. HIF1A, Hypoxia-inducible factor 1-alpha; DNA pol I, DNA polymerase I; DA, Diacridine intercalator; QA, quinoxaline-acridine asymmetric intercalator.


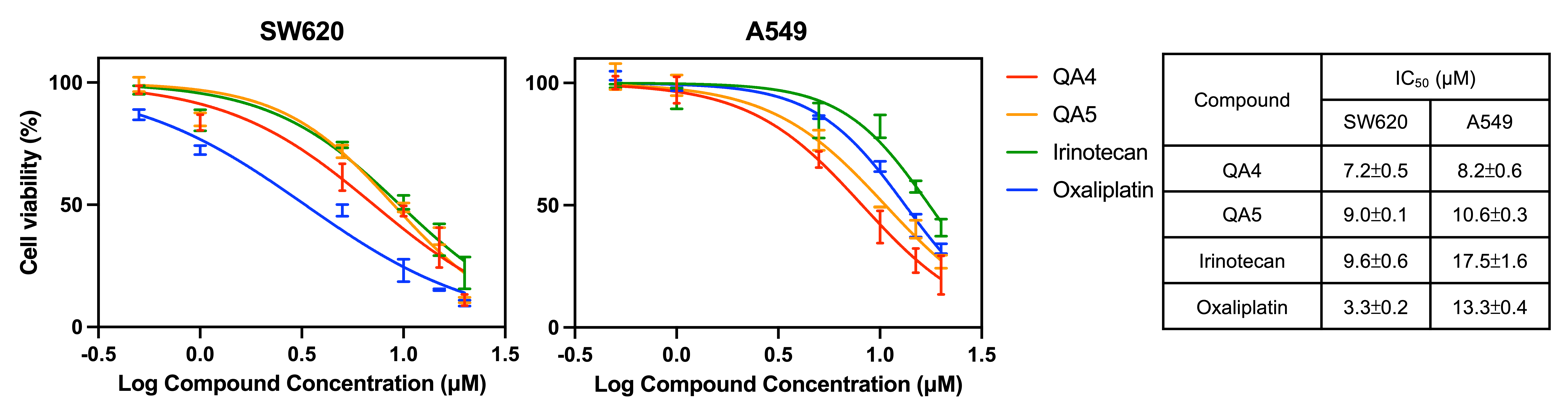


**Figure S8. Cytotoxicity of QA4, QA5, Irinotecan, and Oxaliplatin on SW620 and A549 cancer cells.** The IC_50_ values for QA4, QA5, Irinotecan, and Oxaliplatin were determined based on their effects on the proliferation of SW620 and A549 cancer cells after 48 hours of treatment. Data are derived from three independent experiments. The dose-response curves illustrate the cytotoxic effects of each compound at different concentrations, with cell viability represented as mean ± SD.


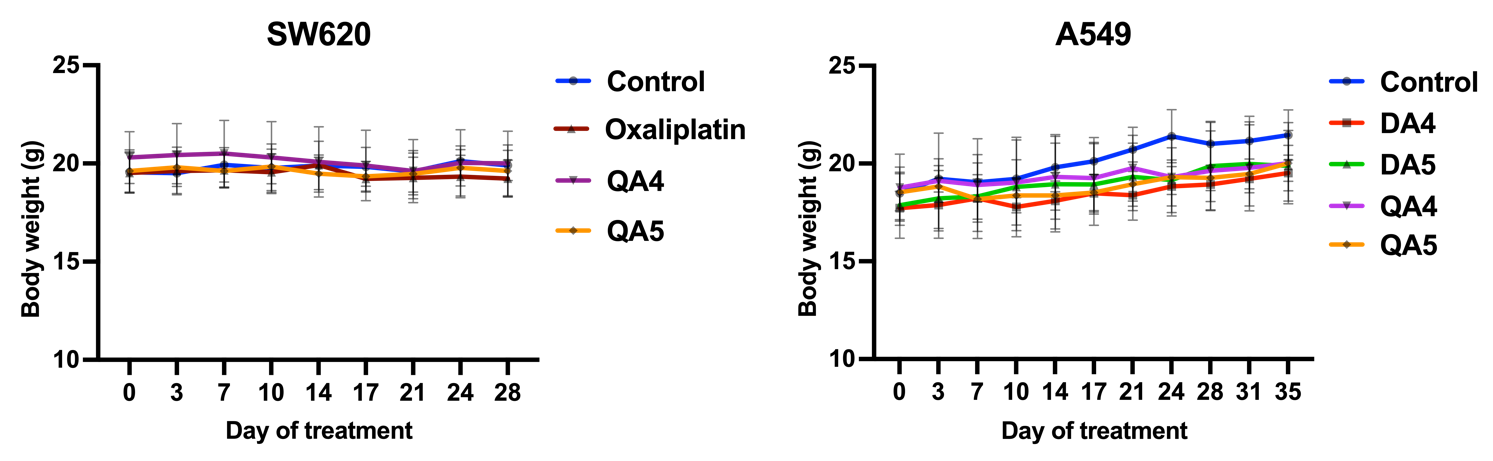


**Figure S9. The body weight change of SW620 and A549 bearing mice with QA4 and QA5 treatment.** Mice were administered 1.5 mg/kg of oxaliplatin and 1.2 mg/kg of DA4, DA5, QA4, and QA5 over a period of 28 days for SW620 tumor-bearing mice and 35 days for A549 tumor-bearing mice.


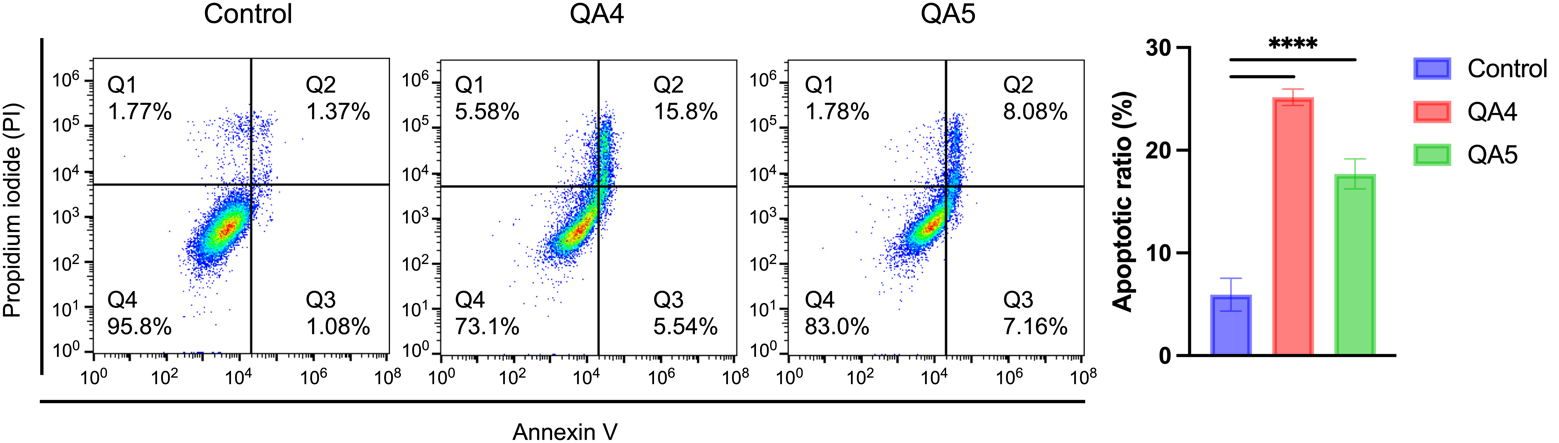


**Figure S10.** **Apoptosis analysis of SW620 cells treated with QA4 and QA5.** SW620 cells were treated with QA4 or QA5 (50 μM) for 24 h and stained with Annexin V-FITC and propidium iodide (PI), followed by flow cytometry analysis. Both QA4 and QA5 increased the apoptotic cell population compared with vehicle control, with QA4 showing a stronger effect. Data are representative of three independent experiments. Bars represent mean ± SD. (using a one-way ANOVA, *, p < 0.05, **, p < 0.01, ***, p < 0.001, ****, p < 0.0001).


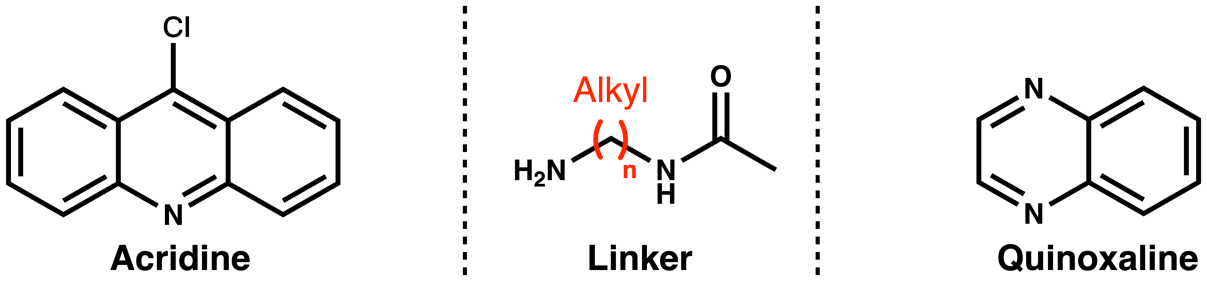


**Figure S11. Bi-targeting asymmetric bis-intercalators.** Chemical scheme and structures of asymmetric acridine and quinoxaline chromophores.


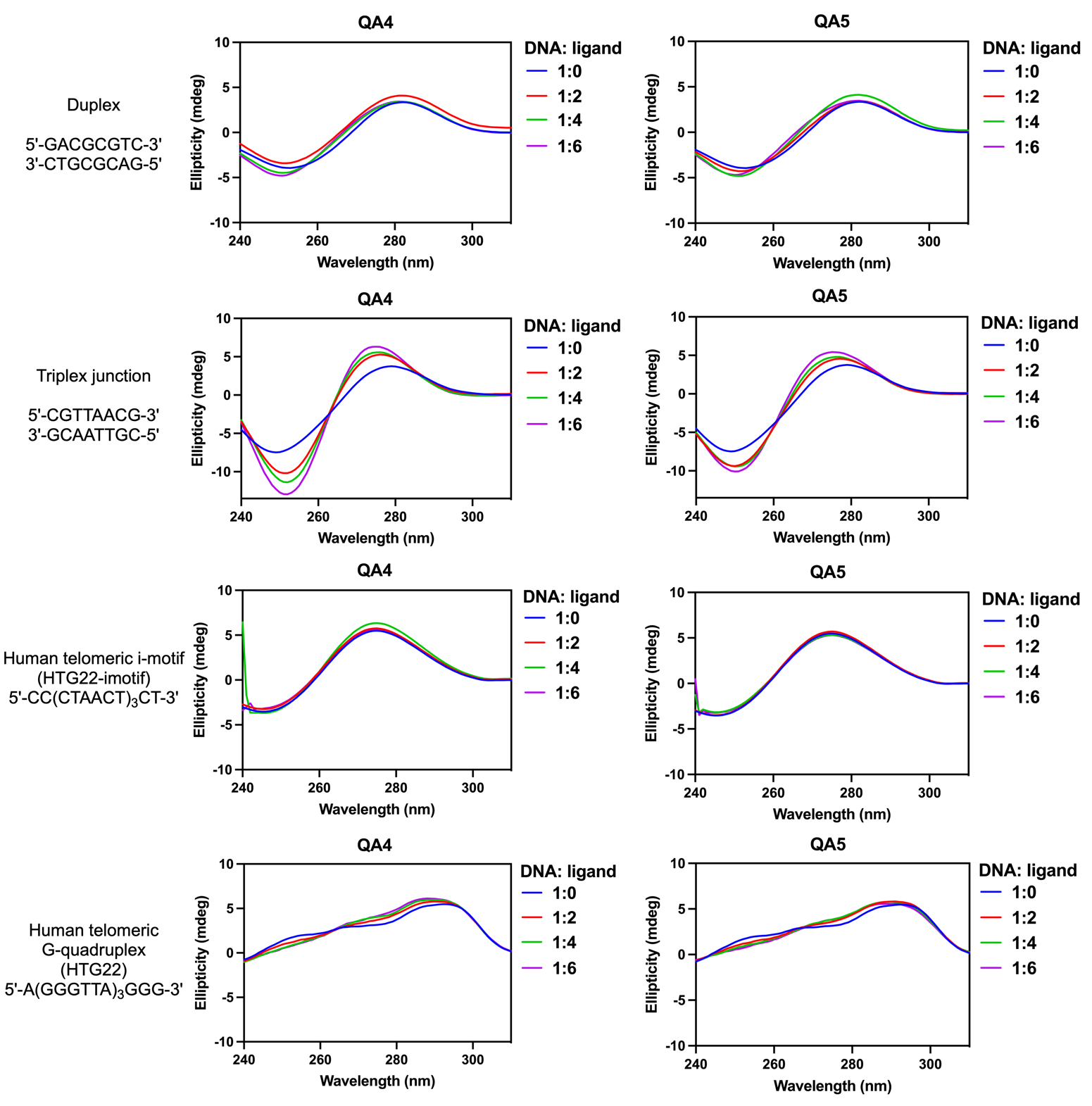


**Figure S12. Circular dichroism (CD) spectroscopic analysis of QA compounds with different DNA secondary structures.** CD spectra were recorded for the duplex control sequence d(GACGCGTC)₂, the junction-forming sequence d(CGTTAACG)₂ used in this study, the human telomeric i-motif-forming sequence, and the human telomeric G-quadruplex-forming sequence (HTG, hybrid form), in the presence of increasing DNA-ligand ratios. The characteristic CD signatures of the structures were consistent with previously reported features, including a positive peak near 280 nm for B-form duplex DNA, a negative peak around 260 nm, and a positive peak near 280 nm for i-motif DNA, and a positive peak near 290 nm with a shoulder at 265 nm for the hybrid telomeric G-quadruplex. Experiments were performed using 20 µM DNA in buffer containing 20 mM sodium cacodylate (pH 7.3), 100 mM KCl, and 5 mM MgCl_2_. Upon addition of QA compounds, the triplex-junction architecture exhibited pronounced ellipticity changes, particularly an enhancement of the positive band near 280 nm together with a deepening of the negative band around 250 nm, whereas only minor spectral variations were observed for the canonical duplex, i-motif, and G-quadruplex structures.


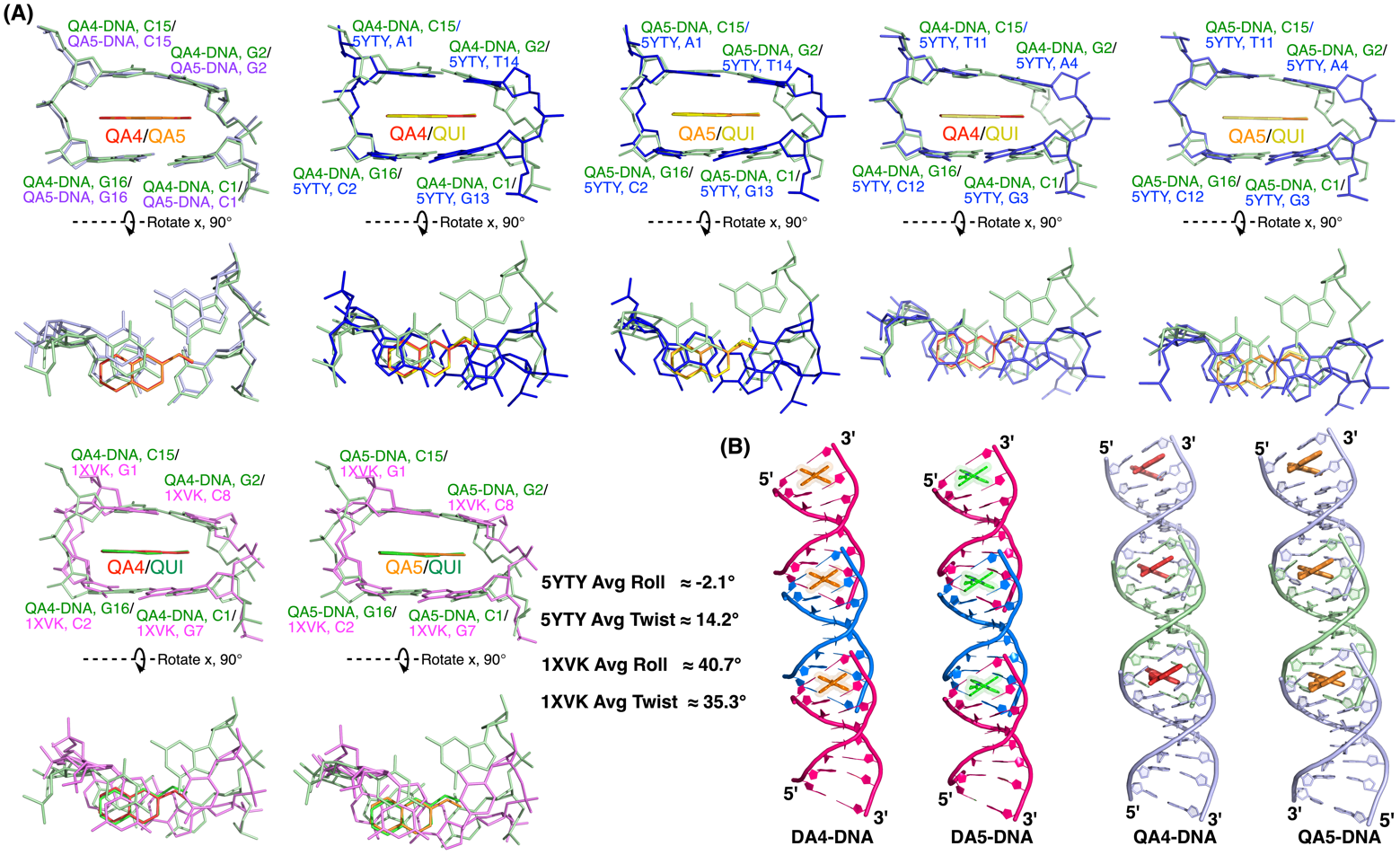


**Figure S13.** **Superimposition of quinoxaline intercalation sites between QA4-DNA, QA5-DNA, PDB: 5YTY, and PDB: 1XVK. (A)** Structural superimposition of the quinoxaline moieties (shown in sticks) intercalated at the C7pG8/C9pG10 site in QA4- and QA5-bound DNA. Superimposition of quinoxaline moieties (shown as sticks) intercalated at the GpT/ApC and GpA/TpC step (PDB: 5YTY), compared with the intercalation sites observed in QA4-DNA and QA5-DNA complexes. Superimposition of quinoxaline moieties (shown as sticks) intercalated at the GpC/GpC step (PDB: 1XVK), compared with the intercalation sites observed in QA4-DNA and QA5-DNA complexes. Different structures are color-coded for clarity. Both side and top views are provided for clear visualization of intercalation modes. **(B)** DA4-DNA, DA5-DNA, QA4-DNA, and QA5-DNA complexes, showing the QA4- and QA5-induced bis-intercalation of the overall DNA structure. Differences in base-pair spacing reflect variations in intercalation-induced rise and roll parameters rather than crystallographic inconsistencies.

**Supplementary Tables**

**Table S1.** Crystallographic and statistical data of CGTTAACG ligand-free DNA, and CGTTAACG with QA4 and QA5 complexes are presented in this study.

| **Data collection statistics** | | | |
| --- | --- | --- | --- |
|  | **d(CGTTAACG)_2_** | **d(CGTTAACG)_2_-QA4** | **d(CGTTAACG)_2_- QA5** |
| Abbreviation | **Ligand-free DNA** | **QA4-DNA** | **QA5-DNA** |
| Beamline | NSRRC TLS 15A, Taiwan | NSRRC TPS 07A, Taiwan | NSRRC TPS 07A, Taiwan |
| Detector type | MX300HE | Eiger2 X 16M | Eiger2 X 16M |
| Wavelength | 1.00000 | 0.97626 | 0.97626 |
| Data collection temperature [K] | 100 | 100 | 100 |
| Space group | *P*3_1_12 | *P*2_1_ | *P*2_1_2_1_2_1_ |
| **Cell dimensions** | | | |
| *a*, *b*, *c* [Å] | 45.447, 45.447, 71.352 | 23.817, 40.150, 24.318 | 23.835, 34.948, 47.901 |
| *α*, *β*, *γ* [°] | 90, 90, 120 | 90, 98.8, 90 | 90, 90, 90 |
| Resolution range [Å]* | 30.00-1.61 (1.61-1.67) | 30.00-1.93 (1.93-2.00) | 30.00-1.27 (1.32-1.27) |
| Total reflections | 60899 | 9978 | 64347 |
| Unique reflections | 11151 | 3366 | 10857 |
| Completeness [%]* | 99.7 (100.0) | 95.7 (96.7) | 97.5 (96.8) |
| Mean *I/σ* [I]* | 35.9 (6.2) | 19.2 (12.5) | 22.1 (9.8) |
| *R*-merge [%]* | 0.04 (0.34) | 0.04 (0.14) | 0.06 (0.20) |
| Redundancy* | 5.3 | 3.2 | 6.4 |
| **Refinement statistics** | | | |
| *R*-work/ *R*-free | 0.21/0.25 | 0.26/0.28 | 0.21/0.23 |
| No. of non-solvent atoms | 635 | 369 | 371 |
| No. of solvent atoms | 66 | 20 | 80 |
| Average B-factor [Å^2^] | 23.0 | 31.00 | 16.00 |
| R.m.s.d bond lengths [Å] | 0.01 | 0.01 | 0.01 |
| R.m.s.d bond angles [°] | 0.9 | 2.0 | 1.3 |
| PDB code | 9V87 | 9V88 | 9V89 |

*Outer shell statistics are shown in parentheses.

**Table S2.** DNA torsion angles and sugar pucker of (a) d(CGTTAACG)_2_ (Ligand-free DNA), (b) d(CGTTAACG)_2_ with QA4 (QA4-DNA), (c) d(CGTTAACG)_2_ with QA5 (QA5-DNA) complexes analyzed by using Curves+ offline software. Values of standard A-DNA and B-DNA are given for comparison.

| (a) | Ligand-free DNA | | | | | | | |
| --- | --- | --- | --- | --- | --- | --- | --- | --- |
|  | **α** | **β** | **γ** | **δ** | **ε** | **ζ** | **χ** | **pucker** |
| A-DNA | -65 | 180 | 60 | 81 | -148 | -71 | -160 | C3'*en* |
| B-DNA | -41 | -136 | 40 | 139 | -133 | -157 | -100 | C2'*en* |
| Base number | | | | | | | | |
| C1 | --- | 151.6 | -70.9 | 153.2 | 153.7 | -100.5 | -172.3 | C2'*en* |
| G2 | -66.8 | 118.2 | -175.5 | 144.0 | -164.8 | -111.4 | -101.5 | C2'*en* |
| T3 | -64.8 | 157.2 | 62.3 | 103.5 | -171.2 | -109.6 | -125.7 | C1'*ex* |
| T4 | -54.8 | 156.5 | 63.7 | 129.0 | 177.1 | -99.0 | -116.0 | C2'*en* |
| A5 | -60.3 | -168.8 | 46.6 | 150.6 | -158.2 | -114.0 | -107.3 | C2'*en* |
| A6 | -48.8 | 157.9 | 39.1 | 108.5 | -162.2 | -102.6 | -127.2 | O1'*en* |
| C7 | -48.4 | 162.0 | 43.1 | 128.8 | -148.8 | -82.2 | -111.1 | C1'*ex* |
| G8 | -59.8 | -170.8 | 53.0 | 101.0 | --- | --- | -61.9 | C2'*ex* |
| *---* | --- | --- | --- | --- | --- | --- | --- | --- |
| *G10* | --- | -112.5 | 3.1 | 155.9 | -178.1 | -92.8 | -90.7 | C3'*ex* |
| *T11* | -69.5 | 160.7 | 64.3 | 96.4 | -173.3 | -104.0 | -130.5 | O1'*en* |
| *T12* | -47.9 | 158.2 | 57.1 | 135.2 | -175.8 | -106.4 | -114.0 | C2'*en* |
| *A13* | -65.6 | -162.7 | 44.3 | 143.9 | -176.2 | -91.8 | -103.7 | O3'*ex* |
| *A14* | -51.8 | 167.9 | 36.9 | 119.4 | -160.1 | -115.3 | -120.5 | C1'*ex* |
| *C15* | -54.4 | 158.3 | 48.4 | 125.7 | -142.3 | -74.1 | -111.8 | C1'*ex* |
| *G16* | -61.4 | -171.9 | 51.5 | 105.0 | --- | --- | -72.8 | C2'*ex* |

| (b) | QA4-DNA complex | | | | | | | |
| --- | --- | --- | --- | --- | --- | --- | --- | --- |
|  | **α** | **β** | **γ** | **δ** | **ε** | **ζ** | **χ** | **pucker** |
| A-DNA | -65 | 180 | 60 | 81 | -148 | -71 | -160 | C3'*en* |
| B-DNA | -41 | -136 | 40 | 139 | -133 | -157 | -100 | C2'*en* |
| Base number | | | | | | | | |
| C1 | --- | -96.3 | 178.6 | 157.7 | -98.2 | -137.9 | -112.7 | C2'en |
| G2 | -71.6 | 173.7 | 35.4 | 134.7 | 176.3 | -108.3 | -89.4 | C2'en |
| T3 | -39.9 | 169.2 | 37.3 | 115.7 | -176.6 | -94.8 | -117.5 | C1'ex |
| T4 | -51.0 | 168.6 | 44.0 | 119.4 | -163.9 | -104.3 | -110.4 | C1'ex |
| A5 | -55.8 | 175.6 | 44.5 | 145.5 | -157.1 | -110.5 | -90.6 | C2'en |
| A6 | -51.5 | 160.1 | 31.8 | 115.7 | -163.1 | 110.5 | -101.2 | C1'ex |
| C7 | -68.1 | 160.0 | 49.4 | 84.7 | -159.3 | -80.9 | -105.9 | C4'ex |
| G8 | -72.5 | -151.9 | 48.2 | 80.9 | --- | --- | -83.5 | C3'en |
| *C9* | --- | 123.3 | 82.5 | 145.4 | -110.8 | -144.0 | -115.5 | C3'ex |
| *G10* | -69.8 | 178.8 | 44.6 | 138.5 | 175.1 | -95.3 | -95.0 | C2'en |
| *T11* | -54.5 | 166.6 | 52.3 | 110.5 | -162.2 | -106.5 | -123.5 | O4'en |
| *T12* | -44.9 | 163.2 | 38.1 | 115.6 | -172.5 | -99.9 | -112.0 | C2'en |
| *A13* | -26.7 | -178.0 | 12.1 | 151.9 | -174.2 | -100.9 | -83.9 | O4'en |
| *A14* | -52.0 | 175.3 | 31.3 | 121.9 | -162.6 | -116.1 | -95.5 | C2'en |
| *C15* | -37.4 | 145.5 | 36.0 | 100.6 | -157.8 | -89.2 | -110.6 | C4'ex |
| *G16* | -84.9 | -167.5 | 69.1 | 82.9 | --- | --- | -95.7 | C4'ex |

| (c) | QA5-DNA complex | | | | | | | |
| --- | --- | --- | --- | --- | --- | --- | --- | --- |
|  | **α** | **β** | **γ** | **δ** | **ε** | **ζ** | **χ** | **pucker** |
| A-DNA | -65 | 180 | 60 | 81 | -148 | -71 | -160 | C3'*en* |
| B-DNA | -41 | -136 | 40 | 139 | -133 | -157 | -100 | C2'*en* |
| Base number | | | | | | | | |
| C1 | --- | 136.2 | 63.9 | 146.1 | -94.6 | -177.5 | -119.9 | C2'en |
| G2 | -70.4 | 165.0 | 42.7 | 138.6 | 178.8 | -97.0 | -107.1 | C2'en |
| T3 | -55.5 | 170.3 | 55.3 | 118.1 | -170.5 | -94.8 | -122.0 | C1'ex |
| T4 | -66.7 | 172.1 | 50.7 | 106.2 | -161.7 | -101.8 | -116.8 | O1'en |
| A5 | -54.1 | 165.5 | 53.4 | 139.9 | -177.8 | -97.5 | -93.7 | C2'en |
| A6 | -59.6 | -177.8 | 37.7 | 123.3 | -160.7 | -92.2 | -98.9 | C1'ex |
| C7 | -66.7 | 162.7 | 47.0 | 92.7 | -164.1 | -84.8 | -107.4 | O1'en |
| G8 | -89.8 | -164.6 | 72.7 | 61.8 | --- | --- | -96.4 | C4'ex |
| *C9* | --- | -98.6 | 152.9 | 147.7 | -113.5 | -145.0 | -115.5 | C2'en |
| *G10* | -68.2 | -171.8 | 48.5 | 147.1 | 176.7 | -102.5 | -80.5 | C3'ex |
| *T11* | -61.2 | 167.6 | 56.3 | 105.8 | 177.2 | -97.5 | -119.2 | O1'en |
| *T12* | -54.2 | 171.4 | 50.7 | 115.6 | -164.3 | -97.0 | -117.6 | C1'ex |
| *A13* | -54.8 | 173.4 | 47.8 | 143.7 | -174.9 | -98.2 | -88.5 | C2'en |
| *A14* | -69.8 | 178.8 | 42.2 | 127.3 | -141.2 | -135.7 | -97.8 | C1'ex |
| *C15* | 75.3 | -126.9 | -150.2 | 84.7 | -133.4 | -73.9 | -144.7 | C3'en |
| *G16* | -66.6 | -151.8 | 57.4 | 91.4 | --- | --- | -86.3 | C4'ex |

**Table S3.** DNA base pair and base pair step parameter of (a) d(CGTTAACG)_2_ (Ligand-free DNA), (b) d(CGTTAACG)_2_ with QA4 (QA4-DNA), (c) d(CGTTAACG)_2_ with QA5 (QA5-DNA) complexes analyzed by using 3DNA 2.0 offline software.

| (a) | Ligand-free DNA | | | | | | |
| --- | --- | --- | --- | --- | --- | --- | --- |
|  | Base pair parameter | | | | | | |
|  |  | **Buckle (°)** | **Propeller (°)** | **Opening (°)** | **Shear (Å)** | **Stretch (Å)** | **Stagger (Å)** |
| Base-pair | **Base-pair identity** | | | | | | |
| C1-G16 | 1 | --- | --- | --- | --- | --- | --- |
| G2-C15 | 2 | -7.3 | 2.5 | -1.9 | -0.2 | -0.2 | -0.6 |
| T3-A14 | 3 | 6.1 | -2.6 | -2.5 | 0.2 | -0.2 | 0 |
| T4-A13 | 4 | 12.8 | -15.3 | 8.0 | -0.6 | 0.1 | -0.3 |
| A5-T12 | 5 | -11.7 | -18.2 | 0.2 | 0.1 | -0.2 | -0.2 |
| A6-T11 | 6 | -6.8 | -9.8 | 1.5 | 0 | -0.1 | 0.2 |
| C7-G10 | 7 | 4.3 | 2.6 | -0.1 | 0.2 | -0.1 | -0.1 |
| G8-C9 | 8 | --- | --- | --- | --- | --- | --- |
|  | Base pair step parameter | | | | | | |
|  |  | **Slide (Å)** | **Shift (Å)** | **Rise (Å)** | **Roll (°)** | **Twist (°)** | **Tilt (°)** |
| Base-pair step | **Step identity** | | | | | | |
| C_1_G_2_/C_15_G_16_ | 1 | --- | --- | --- | --- | --- | --- |
| G_2_T_3_/A_14_C_15_ | 2 | -0.3 | 0 | 3.1 | 3 | 30.4 | -4.1 |
| T_3_T_4_/A_13_A_14_ | 3 | -0.3 | 0 | 3.1 | -2.5 | 27.9 | 4.9 |
| T_4_A_5_/T_12_A_13_ | 4 | 0.2 | -0.1 | 3.8 | 2 | 50.2 | -1.6 |
| A_5_A_6_/T_11_T_12_ | 5 | -0.2 | 0.1 | 3.2 | -3.2 | 33.1 | -5.5 |
| A_6_C_7_/G_10_T_11_ | 6 | -0.3 | -0.2 | 3.1 | 1.4 | 26.2 | 1.8 |
| C_7_G_8_/C_9_G_10_ | 7 | --- | --- | --- | --- | --- | --- |

| (b) | QA4-DNA complex | | | | | | |
| --- | --- | --- | --- | --- | --- | --- | --- |
|  | Base pair parameter | | | | | | |
|  |  | **Buckle (°)** | **Propeller (°)** | **Opening (°)** | **Shear (Å)** | **Stretch (Å)** | **Stagger (Å)** |
| Base-pair | **Base-pair identity** | | | | | | |
| C1-G16 | 1 | 7.2 | -2.2 | -2.3 | 0.2 | -0.2 | -0.2 |
| G2-C15 | 2 | -17.4 | -3.6 | -2.0 | -0.3 | -0.3 | 0.1 |
| T3-A14 | 3 | -9.6 | -10.6 | 2.8 | -0.6 | -0.2 | 0.4 |
| T4-A13 | 4 | -0.8 | -13.3 | 5.4 | 0 | -0.3 | -0.1 |
| A5-T12 | 5 | 1.4 | -13.0 | 5.0 | 0.1 | -0.2 | 0 |
| A6-T11 | 6 | 7.5 | -8.8 | 3.7 | 0.5 | -0.2 | 0.4 |
| C7-G10 | 7 | 29.6 | -10.3 | 2.1 | 0.6 | -0.3 | -0.7 |
| G8-C9 | 8 | -2.5 | 0.6 | -2.8 | -0.1 | -0.3 | -0.4 |
|  | Base pair step parameter | | | | | | |
|  |  | **Slide (Å)** | **Shift (Å)** | **Rise (Å)** | **Roll (°)** | **Twist (°)** | **Tilt (°)** |
| Base-pair step | **Step identity** | | | | | | |
| C_1_G_2_/C_15_G_16_ | 1 | 1.6 | -1.9 | 7.2 | -1.9 | 25.1 | -12 |
| G_2_T_3_/A_14_C_15_ | 2 | 0 | -0.1 | 3.2 | 4.8 | 25.7 | -4.3 |
| T_3_T_4_/A_13_A_14_ | 3 | 0.2 | -0.1 | 3.1 | 7.6 | 31.4 | 3.4 |
| T_4_A_5_/T_12_A_13_ | 4 | 1.1 | 0 | 3.3 | 13.8 | 36.9 | -0.4 |
| A_5_A_6_/T_11_T_12_ | 5 | 0.2 | 0.2 | 3.2 | 7.6 | 31.8 | -3.0 |
| A_6_C_7_/G_10_T_11_ | 6 | 0.2 | 0 | 2.8 | 1.9 | 26.6 | 10.2 |
| C_7_G_8_/C_9_G_10_ | 7 | 2.0 | 2.1 | 7.3 | 4.6 | 24.9 | 9.3 |

| (c) | QA5-DNA complex | | | | | | |
| --- | --- | --- | --- | --- | --- | --- | --- |
|  | Base pair parameter | | | | | | |
|  |  | **Buckle (°)** | **Propeller (°)** | **Opening (°)** | **Shear (Å)** | **Stretch (Å)** | **Stagger (Å)** |
| Base-pair | **Base-pair identity** | | | | | | |
| C1-G16 | 1 | -0.7 | 22.0 | -2.1 | 0.1 | -0.2 | -0.2 |
| G2-C15 | 2 | -12.7 | -2.2 | -0.8 | -0.3 | -0.1 | -0.1 |
| T3-A14 | 3 | -3.6 | -8.3 | 1.5 | -0.2 | -0.1 | 0.2 |
| T4-A13 | 4 | 1.4 | -15.3 | 6.6 | 0 | -0.2 | -0.3 |
| A5-T12 | 5 | -0.1 | -16.5 | 3.9 | 0.1 | -0.1 | -0.3 |
| A6-T11 | 6 | 5.1 | -10.2 | 3.1 | 0.1 | -0.1 | 0.3 |
| C7-G10 | 7 | 14.4 | 1.6 | -0.5 | 0.2 | -0.1 | -0.1 |
| G8-C9 | 8 | -5.2 | 1.7 | -0.1 | -0.3 | -0.3 | -0.6 |
|  | Base pair step parameter | | | | | | |
|  |  | **Slide (Å)** | **Shift (Å)** | **Rise (Å)** | **Roll (°)** | **Twist (°)** | **Tilt (°)** |
| Base-pair step | **Step identity** | | | | | | |
| C_1_G_2_/C_15_G_16_ | 1 | 1.0 | -2.9 | 6.7 | -7.1 | 22.0 | -15.0 |
| G_2_T_3_/A_14_C_15_ | 2 | -0.3 | -0.5 | 3.1 | 1.9 | 26.2 | -2.8 |
| T_3_T_4_/A_13_A_14_ | 3 | -0.2 | -0.2 | 3.1 | 7.4 | 31.2 | 3.0 |
| T_4_A_5_/T_12_A_13_ | 4 | 1.4 | -0.2 | 3.5 | 12.6 | 35.9 | -0.8 |
| A_5_A_6_/T_11_T_12_ | 5 | -0.2 | 0.3 | 3.1 | 4.9 | 32.2 | -2.3 |
| A_6_C_7_/G_10_T_11_ | 6 | 0 | 0.4 | 3.2 | 7.3 | 23.7 | 4.4 |
| C_7_G_8_/C_9_G_10_ | 7 | 1.7 | 2.4 | 7.1 | -2.6 | 24.5 | 11.3 |

**Supplementary References**

1. Marenich, A.V., Cramer, C.J. and Truhlar, D.G. (2009) Universal Solvation Model Based on Solute Electron Density and on a Continuum Model of the Solvent Defined by the Bulk Dielectric Constant and Atomic Surface Tensions. *The Journal of Physical Chemistry B*, **113**, 6378-6396.
